# A First-Passage Model of Intravitreal Drug Delivery and Residence Time—Influence of Ocular Geometry, Individual Variability, and Injection Location

**DOI:** 10.1167/iovs.65.12.21

**Published:** 2024-10-16

**Authors:** Patricia Lamirande, Eamonn A. Gaffney, Michael Gertz, Philip K. Maini, Jessica R. Crawshaw, Antonello Caruso

**Affiliations:** 1 Wolfson Centre for Mathematical Biology, Mathematical Institute, Andrew Wiles Building, University of Oxford, Oxford, United Kingdom; 2 Pharmaceutical Sciences, Roche Innovation Center Basel, Roche Pharma Research and Early Development, Basel, Switzerland; 3 School of Mathematical Sciences, Queensland University of Technology, Brisbane, Australia

**Keywords:** diffusion, intravitreal drug delivery, first-passage time, large molecule pharmacokinetics, interspecies translation

## Abstract

**Purpose:**

Standard of care for various retinal diseases involves recurrent intravitreal injections. This motivates mathematical modeling efforts to identify influential factors for ocular drug residence time, aiming to minimize administration frequency. We sought to describe the vitreal diffusion of therapeutics in nonclinical species frequently used during drug development assessments. In human eyes, we investigated the impact of variability in vitreous cavity size and eccentricity, and in injection location, on drug disposition.

**Methods:**

Using a first-passage time approach, we modeled the transport-controlled distribution of two standard therapeutic protein formats (Fab and IgG) and elimination through anterior and posterior pathways. Anatomical three-dimensional geometries of mouse, rat, rabbit, cynomolgus monkey, and human eyes were constructed using ocular images and biometry datasets. A scaling relationship was derived for comparison with experimental ocular half-lives.

**Results:**

Model simulations revealed a dependence of residence time on ocular size and injection location. Delivery to the posterior vitreous resulted in increased vitreal half-life and retinal permeation. Interindividual variability in human eyes had a significant influence on residence time (half-life range of 5–7 days), showing a strong correlation to axial length and vitreal volume. Anterior exit was the predominant route of drug elimination. Contribution of the posterior pathway displayed a 3% difference between protein formats but varied between species (10%–30%).

**Conclusions:**

The modeling results suggest that experimental variability in ocular half-life is partially attributed to anatomical differences and injection site location. Simulations further suggest a potential role of the posterior pathway permeability in determining species differences in ocular pharmacokinetics.

The eye is a complex organ that varies significantly in size and shape between different species. In the human eye, individual variations of size and shape are common and can cause various vision conditions. With emmetropia describing the absence of refractive error, myopia is generally characterized by an elongated eye,^1^ with a larger axial length (AL) compared to an emmetropic eye,^2^ while hypermetropia is often associated with a shorter AL.^3^

Approximately one in three people have some form of disease-induced vision impairment by the age of 65.^4^ A common cause of vision loss among the elderly is age-related macular degeneration (AMD), a progressive disease characterized by damage to the macula.^4^ The wet form of AMD is characterized by upregulation of the vascular endothelial growth factor (VEGF), an angiogenic protein^5^ that induces pathological neovascular growth leading to retinal damage.^6^^,^^7^

Among treatment options for wet AMD are intravitreal (IVT) injections of protein therapeutics that bind to VEGF to inhibit its function.^5^ Two standard-of-care therapeutics are ranibizumab, a monoclonal antibody fragment (Fab), and aflibercept, an Fc-fusion protein, with reported hydrodynamic radii (*R*_*h*_) of 3.0 and 5.2 nm, respectively.^8^^,^^9^ The latter is comparable to the macromolecular size of bevacizumab (5.0 nm),^8^ a monoclonal full-length IgG1 antibody^10^ that is used off-label for the treatment of choroidal neovascularization. These antibodies are administered through IVT injections, and regular administrations were shown to improve visual acuity outcome in the majority of patients.^11^^–^^13^ However, IVT drugs exhibit suboptimal drug retention, with clinical studies reporting the ocular half-lives of less than 10 days.^14^^,^^15^

IVT injections in the human eye are not targeting a specific injection site within the vitreous chamber.^16^ Broad guidelines have been specified; for example, the needle tip should be inserted more than 6 mm aiming at the center of the eye^17^ with the bevel facing upward,^18^ which provides less than a precise target for the injection site. It is also advised to deliver the dosing formulation gently into the vitreous cavity with a slow injection, in order to avoid jet formation and excessive cavitary flow.^17^^,^^18^ In general, procedures are specified with focus on preventing mechanical damage and infections, with seemingly less attention paid to the potential impact on drug absorption or ocular residence time.^17^^,^^19^ However, chronic treatment places a burden on patients and health care systems in terms of resources and procedural risks.^20^^,^^21^ Moreover, it is estimated that the majority of the injected drug is eliminated through the anterior pathway via aqueous humor turnover, with a small proportion permeating the retina (posterior elimination pathway), despite being the target site of action.^22^^,^^23^ This motivates investigation of drug residence time in the eye due to differences in eye shape and size, drug hydrodynamic radii, and injection locations.

The translation of results across nonclinical species and patients is crucial for the effective design and characterization of drug candidates. Species commonly used in ocular pharmacokinetic (PK) or pharmacodynamic (PD) studies are the rabbit,^9^^,^^24^^–^^29^ cynomolgus monkey,^30^^–^^32^ and rat,^33^^,^^34^ with fewer studies reported in pig^35^^–^^37^ and mice.^38^^,^^39^ Previous studies have demonstrated the role of diffusion in determining the vitreal elimination rate of IVT macromolecules, with PK experiments showing a dependence of ocular half-life on both the molecular size and eye size.^8^^,^^9^^,^^40^ In particular, in Caruso et al. (2020),^8^ the ocular half-life was expressed as a linear function of Rh×r vit 2, with *r*_vit_ the vitreal radius (under the assumption of a spherical vitreous chamber), and with a species-specific slope. With the slopes varying between species, it was postulated that other factors than diffusion distance must explain the species-specific half-lives.^8^ For example, the experimental half-life obtained for a given molecule or molecular size is larger in pigs than in humans and in rabbits than in monkeys, although the respective vitreous volumes are smaller. Caruso et al.^8^ and Crowell et al.^40^ postulated that the observed species-specific PK could result from differences in ocular shape and eccentricity or in the contribution of the posterior pathway to drug elimination, among other factors. This motivates modeling efforts in investigating these aspects to support the translational characterization of novel drug candidates.

Scaling relationships between species have been previously established^41^ under the assumption of spherical vitreous chambers, implying simplified ocular anatomies. More anatomically faithful models are also available, such as the computational fluid dynamics works of Missel^42^ and Lamminsalo et al.^43^^,^^44^ that describe both the posterior and anterior segments in great detail. Besides the complexity and computational cost inherent to such models, the rate of egress of material from the vitreous remains the rate-limiting factor and main determinant of ocular PK, so studying the distribution within the vitreous cavity and at its interfaces in more detail is key.

As defined in the random walk field, the first passage time is a random variable describing how long it takes for a random walker to reach a given target site.^45^^,^^46^ Its expected value is called the mean first passage time (MFPT). The MFPT has been described as an effective measure of diffusive transport.^47^^–^^49^ Recent applications in mathematical biology have been diverse, including applications to animal movement in heterogeneous landscapes,^50^ receptors in the synaptic membrane,^51^ and drug molecules crossing the mucus–epithelium interface.^52^ In ocular modeling, an approximation of the MFPT was previously used to define the vitreal diffusion time, the average time for a particle to diffuse from the center of a sphere to its surface, with the vitreous chamber modeled as a sphere in Hutton-Smith et al.^41^ However, the first-passage problem was not explicitly solved. In the present MFPT modeling framework, we considered isotropic diffusion and the absence of convection in the distribution of the injected ocular drug, justified by the slow injection of the drug and by previous experiments supporting the absence of flow in the vitreous chamber.^53^^–^^57^ The MFPT is a measure of the residence time (which does not depend on initial drug concentration), and its framework can be extended to quantify the drug elimination using the distribution of exits, the multidimensional analogue of the splitting probabilities.^47^^,^^48^^,^^58^ This can be used to quantify the amount of drug leaving through each elimination pathway and their relative importance.

This research aims to investigate the influence of ocular size and shape, interindividual variability, drug molecular features, and injection location on the vitreal kinetics and residence time of IVT macromolecules. We further aim to study whether interspecies differences in vitreous chamber geometry may explain the different pharmacokinetics observed experimentally. To this end, we develop a mathematical model of vitreal drug diffusion based on the first-passage time methodology, deriving equations for the MFPT, the conditional MFPT, and the drug elimination distribution for two standard molecular formats of IVT therapeutics, namely, Fab and IgG. Using literature datasets to construct realistic three-dimensional (3D) geometries, we model human emmetropic, myopic, and hypermetropic eyes for studying the influence of ocular shape on drug retention. We also model the anatomy of the vitreous chamber in the mouse, rat, rabbit, and cynomolgus monkey, aiming to improve the translational understanding of PK in support of drug discovery and development. In order to assess the influence of spatial parameters, we compare the residence time in the different vitreous chamber geometries and derive a scaling relationship between the MFPT, vitreal volume, and AL. We also assess the dependence of retinal absorption on the spatial parameters describing the ocular geometries and on the site of injection within the vitreous cavity, as well as identify the dominant elimination pathway kinetics using the conditional MFPT.

## Methods

### Ocular Geometry

**Figure 1.**
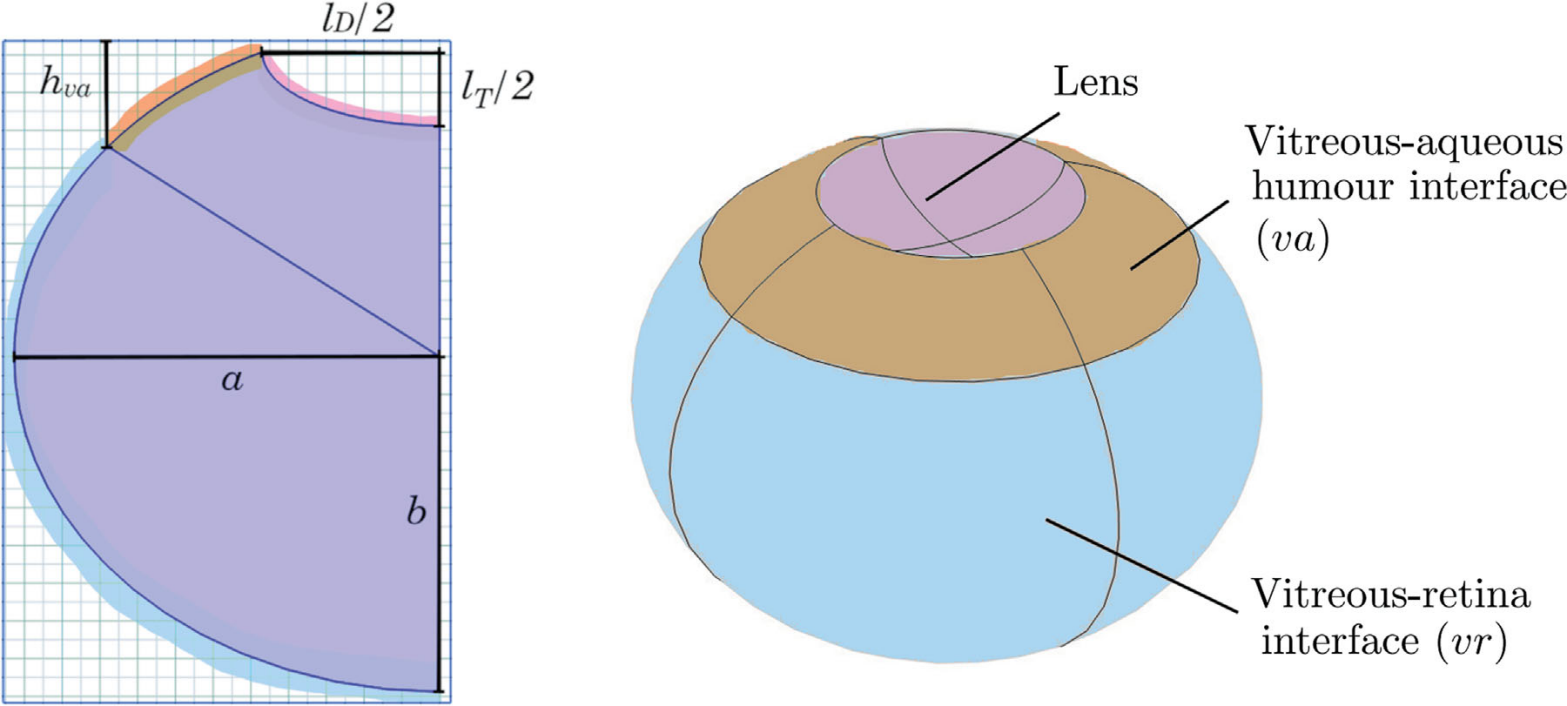
Plane geometry used in the axial rotation to define the 3D ocular model for the human eye, where *a* and *b* are the semi-major and semi-minor axes of the vitreous chamber ellipse, *l*_*D*_ and *l*_*T*_ are the lens diameter and thickness, and *h*_*va*_ is the height of the vitreous–aqueous humor interface. The vitreous–lens interface is identified in pink, the vitreous–aqueous humor interface in orange, and the vitreous–retina interface in blue. Parameters not shown: *l*_*p*_, the proportion of the lens thickness within the vitreous chamber ellipse, and *V*_*vit*_ and *A*_*ret*_, the volume of the vitreous humor and the area of the retinal surface.

The 3D models of the vitreous chamber were built for human and relevant nonclinical species. The posterior cavity was assumed to be an oblate spheroid, obtained by the rotation of an ellipse around its minor axis, which was collinear with the optical axis. The lens protruding into the vitreous humor was similarly defined. The difference between the two determined the vitreous chamber (Fig. 1). Three interfaces were defined, corresponding to the vitreous–lens, vitreous–aqueous humor, and vitreous–retina boundaries. Anatomically, the vitreous–retina interface corresponds to the surface covered by the inner limiting membrane (ILM) and delimited by the ora serrata, and the vitreous-aqueous humor interface corresponds to the zonular fibers and the space of Petit. The parameters used to build the 3D geometries are defined in Figure 1, and the details of the construction of the geometries are summarized in Supplementary S1.2.

**Figure 2.**
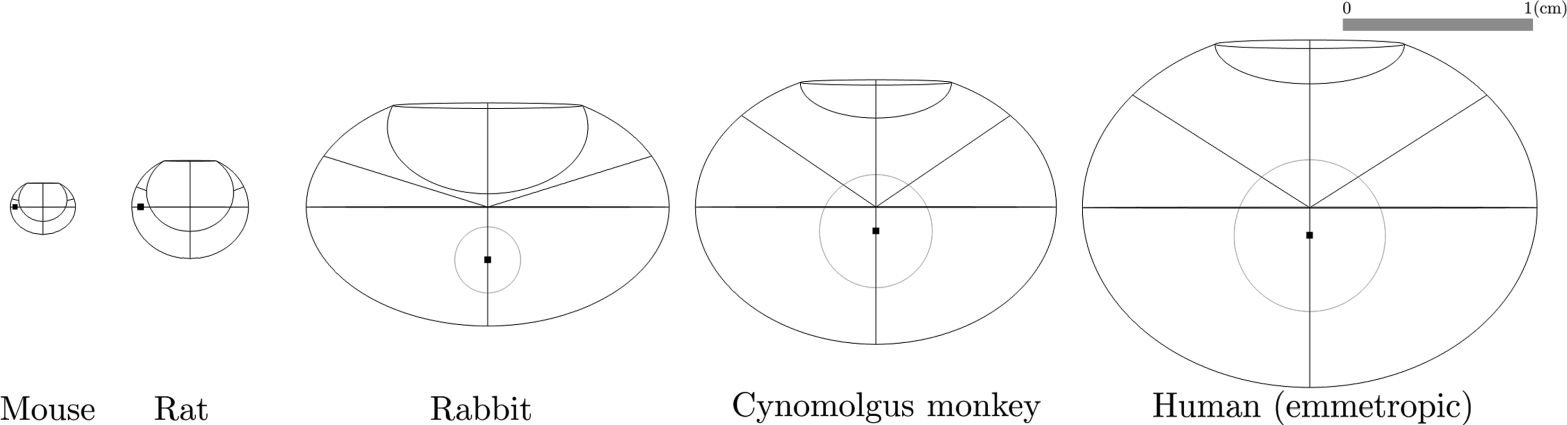
Cross sections of the ocular geometries built using parameters in Table 1 and based on Figure 1, in relative scale. The black bullet represents the injection point *P*_*m*_, and the gray circle in the rabbit, cynomolgus monkey, and human eye models represents the injection location region considered in the ocular half-life (*t*_1/2_) analysis.

**Figure 3.**
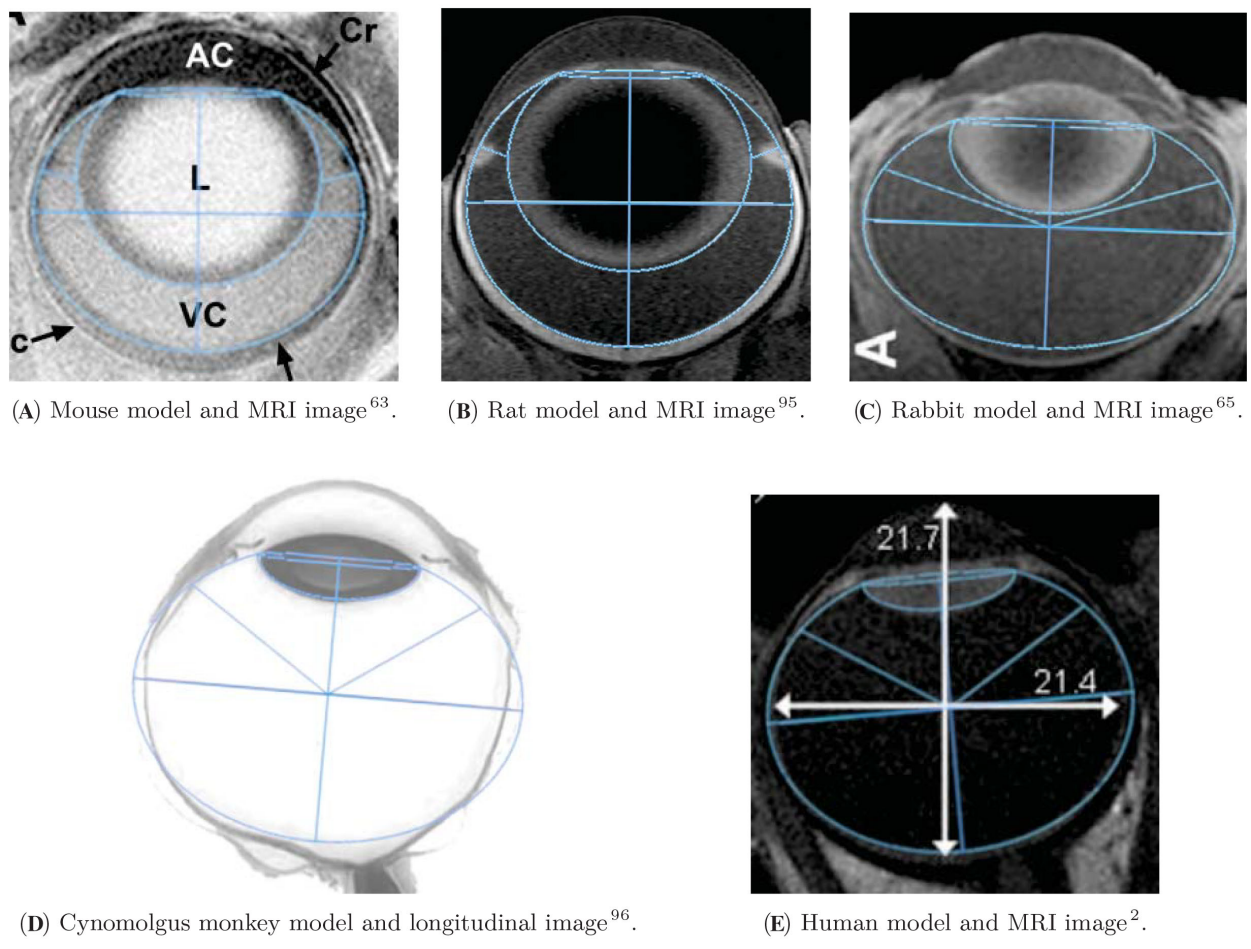
(**A**–**C**, **E**) Superimposition of the ocular models (*blue lines*) and in vivo MRI images. The geometries in Figure 2 were scaled to the anatomical features of the MRI images with a constant aspect ratio. (**D**) No MRI images of the cynomolgus monkey eye could be found in the literature; therefore, a light micrograph of a longitudinal section (*grayscale*) was used instead. Annotations in (**A**, **C**, **E**) originate from the source images. (**C**) Reprinted by permission of Taylor & Francis Ltd. from Sawada T, Nakamura J, Nishida Y, Kani K, Morikawa S, Inubushi T. Magnetic resonance imaging studies of the volume of the rabbit eye with intravenous mannitol. *Curr Eye Res.* 2002;25(3):173–177. (**D**) Reprinted by permission of Sage Publications from Short BG. Safety evaluation of ocular drug delivery formulations: techniques and practical considerations. *Toxicol Pathol.* 2008;36(1):49–62.

A literature search was conducted to collect the anatomical dimensions of human eyes as well as those of the cynomolgus monkey, rabbit, rat, and mouse. Insufficient anatomical information prevented inclusion of the pig or minipig among modeled species. A summary of the literature data is provided in Table 1. The measurements were performed by various methods, including magnetic resonance imaging (MRI), optical coherence tomography, ultrasound biometry, Scheimpflug photography, and direct measurements of postmortem fixed eyes. The parameter values for the model geometries are also summarized in Table 1 and detailed in Supplementary S1.2. A cross section of the model for each species is displayed in relative scale in Figure 2. We verified the anatomical accuracy of the geometries by comparison with experimental measurements of vitreous volumes and retinal areas (*V*_*vit*_ and *A*_*ret*_ in Table 1) and with in vivo MRI images obtained from the literature, as illustrated in Figure 3.

**Table 1.** Literature Values of Ocular Geometry Measures and Ocular Model Dimensions

	Mouse		Rat		Rabbit	
Parameter	Literature Value	Model Value	Literature Value	Model Value	Literature Value	Model Value
*a* (cm)	0.161–0.163^63^	0.1618	0.286–0.293^64^	0.2895	0.88–0.92^65^	0.90
*b* (cm)	0.127–0.139^66^	0.1355	0.253–0.257^64^	0.255	0.566–0.611^67^	0.588
*l*_*D*_ (cm)	0.223–0.245^68^	0.240	0.423–0.51^64^^,^^69^^,^^70^	0.432	0.971–1.02^71^	0.995
*l*_*T*_ (cm)	0.197–0.241^63^^,^^66^^,^^68^	0.216	0.371–0.457^64^^,^^69^^,^^72^	0.387	0.606–0.697^67^^,^^73^	0.66
*l*_*p*_ (%)		99		99		66
*h*_*va*_ (cm)		0.05		0.07		0.238
*V*_vit_ (ml)	0.0044–0.012^39^^,^^74^^–^^76^	0.00842	0.0505–0.0543^77^	0.0518	1.15–1.80^73^^,^^78^^,^^79^	1.71
*A*_ret_ (cm^2^)	0.134–0.190^80^^–^^82^	0.188	0.65–0.8^83^^,^^84^	0.667	4–6^85^	5.44
		**Cynomolgus Monkey**		**Human**		
**Parameter**		**Literature Value**	**Model Value**	**Literature Value**		**Model Value**

*a* (cm)		0.855–0.915*	0.895	1.03–1.25^2^^,^^60^^,^^61^		1.1275
*b* (cm)		0.623–0.733^86^^,^^87^	0.678	0.816–1.07^2^^,^^60^^,^^61^^,^^88^		0.889
*l*_*D*_ (cm)		0.73–0.79^89^	0.75	0.88–0.985^71^^,^^89^^,^^90^		0.939
*l*_*T*_ (cm)		0.288–0.403^73^^,^^86^^,^^87^	0.351	0.391–0.564^71^^,^^88^^,^^90^		0.3909
*l*_*p*_ (%)			50			50
*h*_*va*_ (cm)			0.163			0.251
*V*_vit_		2.0–2.3^73^	2.20	3.58–6.38^60^^,^^61^		4.60
*A*_ret_ (cm^2^)		5.8–9.2^91^	6.91	10.12–13.63^92^^–^^94^		11.0

The derivation of the model parameters is detailed in Supplementary S1.2. *Estimated from *V*_*vit*_.

To investigate their influence on ocular PK, different injection site locations within the vitreous chamber were considered. Under the assumption that IVT injections target the central vitreous, equidistantly from the retina and the posterior surface of the lens, a region of interest was defined around the midpoint (*P*_*m*_) of the vitreous chamber depth. For simplicity, a sphere of diameter corresponding to half of the vitreous chamber depth was defined (Fig. 2). The resulting volume encompasses the vitreous core, arguably representing a conservative estimate for the possible locations of IVT delivery. This injection region was employed to assess the impact on ocular half-life, *t*_1/2_, in human, cynomolgus monkey, and rabbit eyes. In rodents, the lens occupies a significant portion of the posterior cavity (Fig. 3), giving the vitreous chamber a distinctive crescent shape, for which it is more difficult to define the center and reach it with an injection needle. Therefore, in the rat and mouse model, *P*_*m*_ was defined as the midpoint between the retina and the lens, along the vitreous diameter (Fig. 2).

**Figure 4.**
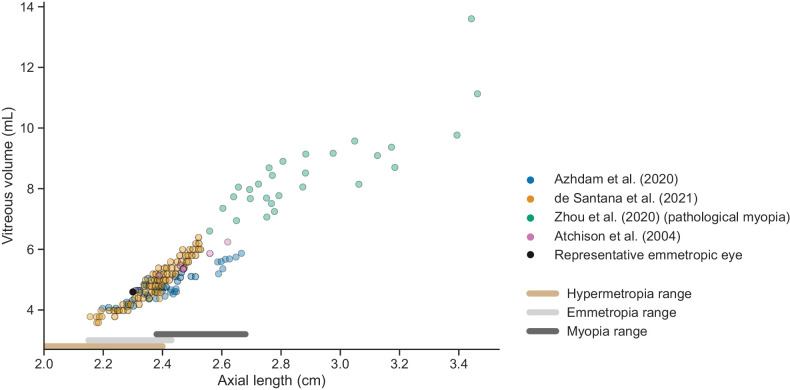
Literature measurements of vitreous volume and axial length (AL) in 155 human eyes, used to build the ensemble of human eye models. The hypermetropia, emmetropia, and myopia ranges are identified using definitions by Strang et al.^3^ and Atchison et al.^2^ *Black bullet*: emmetropic human eye model corresponding to the geometry of Figure 2.

Additionally, an ensemble of 155 human eye models was built based on AL and vitreous volume measurements obtained from the literature (Fig. 4). The measurements were collected by MRI,^2^^,^^59^ optical biometry and vitrectomy,^60^ and computed tomography (CT) scan.^61^ The AL data collectively cover the range associated with hypermetropic, emmetropic, and myopic eyes,^2^^,^^3^ and include data for pathological myopia, described as a refractive error of −8 diopters or lower.^59^^,^^62^ Using the vitreous volume and AL, the eye geometries were constructed assuming a constant lens thickness and anterior chamber depth. The reader is referred to Supplementary S1.2 and Supplementary S2 for further details.

### Equations

The equations have been derived by expanding the first-passage time approach^47^^–^^49^^,^^97^^,^^98^ and applying it to vitreal transport.

#### Mean First-Passage Time

The MFPT, $\tau (x_{0})$, for a particle starting at $x_{0}$, satisfies the following partial differential equation (PDE) and boundary conditions:

(1) $$\begin{array}{c} \begin{array}{cccc} -D\;\nabla^{2}\tau \left(x_{0}\right) & = & 1 & \text{for}\;x_{0}\in \Omega ,\\ \nabla \tau (x_{0})\cdot n & = & 0 & \text{for}\;x_{0}\in \partial \Omega_{vl},\\ -D\;\nabla \tau (x_{0})\cdot n & = & \kappa_{va}\;\tau \left(x_{0}\right) & \text{for}\;x_{0}\in \partial \Omega_{va},\\ -D\;\nabla \tau (x_{0})\cdot n & = & \kappa_{vr}\;\tau \left(x_{0}\right) & \text{for}\;x_{0}\in \partial \Omega_{vr}, \end{array} \end{array}$$

where n is the outward normal and with parameters defined in Table 2, for the vitreal region Ω and associated lens, anterior and retinal boundaries ∂Ω_*vl*_, ∂Ω_*va*_, and ∂Ω_*vr*_ respectively, as illustrated in Figure 1.

**Table 2.** Definition of the Drug-Dependent Parameters for the Fab and IgG Molecular Formats

Drug-Dependent Parameters	Fab	IgG
Diffusion coefficient (*D*)	1.07 × 10^−6^ cm^2^/s [8]	0.64 × 10^−6^ cm^2^/s [8]
Permeability of vitreous–aqueous humor interface (*k*_*va*_)	1.91 × 10^−5^ cm/s [99]	0.874 × 10^−5^ cm/s [99]
Permeability of vitreous–retina interface (*k*_*vr*_)	1.81 × 10^−7^ cm/s [99]	1.19 × 10^−7^ cm/s [99]

The MFPT can be linked to the ocular half-life *t*_1/2_ (i.e., the time required for a quantity that is exponentially decaying to fall to half of its initial value). In this context, it characterizes the rate at which the drug is cleared from the vitreous chamber. Under the assumption that the drug concentration inside the vitreous is decreasing exponentially after an IVT injection (see Supplementary S1.1), the MFPT corresponds to the inverse of the decay rate, and we obtain the relation:

(2) $$\begin{array}{c} t_{1/2}\left(x_{0}\right)=\tau \left(x_{0}\right)\ln\left(2\right) \end{array}$$

for an injection at $x_{0}$.

#### Drug Elimination and Conditional Mean First-Passage Time

Let $\pi_{va}\left(x_{0}\right)$ be the proportion of drug leaving through the vitreous–aqueous humor interface (∂Ω_*va*_) and $\pi_{vr}\left(x_{0}\right)$ the proportion leaving through the vitreous–retina interface (∂Ω_*vr*_), where $x_{0}$ is the initial position. Note that these are the only regions in the model where molecules can exit and, therefore, the proportions sum to 1. We define $T_{va}\left(x_{0}\right)$ as the MFPT conditional on drug molecules leaving through ∂Ω_*va*_ and $T_{vr}\left(x_{0}\right)$ the conditional MFPT for molecules leaving through ∂Ω_*vr*_, both functions of the molecules' initial position $x_{0}$. The proportion of drug exiting through ∂Ω_*vr*_, $\pi_{vr}\left(x_{0}\right)$, satisfies the PDE system^48^^,^^49^^,^^98^:

(3) $$\begin{array}{c} \begin{array}{cccc} \nabla^{2}\pi_{vr}\left(x_{0}\right) & = & 0 & \text{for}\;x_{0}\in \Omega ,\\ \nabla \pi_{vr}\left(x_{0}\right)\cdot n & = & 0 & \text{for}\;x_{0}\in \partial \Omega_{vl},\\ -D\;\nabla \pi_{vr}\left(x_{0}\right)\cdot n & = & \kappa_{va}\;\pi_{vr}\left(x_{0}\right) & \text{for}\;x_{0}\in \partial \Omega_{va},\\ -D\;\nabla \pi_{vr}\left(x_{0}\right)\cdot n & = & -\kappa_{vr}+\kappa_{vr}\;\pi_{vr}\left(x_{0}\right) & \text{for}\;x_{0}\in \partial \Omega_{vr}, \end{array} \end{array}$$

and $T_{vr}\left(x_{0}\right)$ satisfies^48^^,^^98^:

(4) $$\begin{array}{c} \begin{array}{cccc} -D\;\nabla^{2}\left[\pi_{vr}\left(x_{0}\right)\;T_{vr}\left(x_{0}\right)\right] & = & \pi_{vr}\left(x_{0}\right) & \text{for}\;x_{0}\in \Omega ,\\ \nabla [\pi_{vr}\left(x_{0}\right)\;T_{vr}\left(x_{0}\right)]\cdot n & = & 0 & \text{for}\;x_{0}\in \partial \Omega_{vl},\\ -D\;\nabla [\pi_{vr}\left(x_{0}\right)\;T_{vr}\left(x_{0}\right)]\cdot n & = & \kappa_{va}\;\left[\pi_{vr}\left(x_{0}\right)\;T_{vr}\left(x_{0}\right)\right] & \text{for}\;x_{0}\in \partial \Omega_{va},\\ -D\;\nabla [\pi_{vr}\left(x_{0}\right)\;T_{vr}\left(x_{0}\right)]\cdot n & = & \kappa_{vr}\;\left[\pi_{vr}\left(x_{0}\right)\;T_{vr}\left(x_{0}\right)\right] & \text{for}\;x_{0}\in \partial \Omega_{vr}. \end{array}\; \end{array}$$

The conditional MFPT for drug molecules leaving through the vitreous–aqueous humor, $T_{va}\left(x_{0}\right)$, was derived following the same method.

### Drug-Dependent Parameters

The drug-dependent parameters present in the PDE systems (Equations (1) to (4)) were set using experimental measures and modeling results found in the literature. The value of the diffusion coefficient *D* is well defined by experimental studies in the literature and was set to *D* = 1.07 × 10^−6^ cm^2^/s and *D* = 0.64 × 10^−6^ cm^2^/s for a Fab and IgG molecular format, respectively.^8^

The permeability parameters κ_*va*_ and κ_*vr*_, for the vitreous–aqueous humor and vitreous–retina interface, respectively, were more difficult to determine. In addition to varying with the drug molecule size, the permeability parameters are reported to vary between different species.^100^ Previous estimates of these parameters for Fab and IgG molecules are summarized in Table 3. To incorporate these estimates into our model, we identified the vitreous–aqueous humor interface as equivalent to the hyaloid membrane described in Hutton-Smith.^99^ For the vitreous–retina interface, we designated its permeability to the lowest permeability between the retinal pigment epithelium (RPE) and the ILM defined in Hutton-Smith et al.^23^^,^^99^^,^^101^ As there was only one estimate for the vitreous–aqueous humor permeability, we set κ_*va*_ = 1.91 × 10^−5^ cm/s, and we used the estimates from the same reference^99^ to set κ_*vr*_ = 1.81 × 10^−7^ cm/s, both for a Fab molecule. For the IgG molecule, we followed the same steps and set κ_*va*_ = 0.874 × 10^−5^ cm/s and κ_*vr*_ = 1.19 × 10^−7^ cm/s, using the estimations from Hutton-Smith.^99^ We note that the permeability parameters in Table 3 have a high degree of uncertainty, as they cannot be measured directly, and were estimated by fitting mathematical models to rabbit data.

**Table 3.** Literature Values of Ocular Permeabilities

Permeability	Fab	IgG	Source
RPE permeability (× 10^−7^ cm/s)	2.60 (1.36, 4.04)	1.84 (1.08, 2.36)	^23^
	2.63		^101^
	2.48 (2.2, 5.35)	2.31 (1.76, 2.98)	^99^
ILM permeability (× 10^−7^ cm/s)	1.88 (1.13, 2.81)	1.7 (0.912, 2.32)	^23^
	1.89		^101^
	1.81 (1.25, 2.44)	1.19 (1.12, 1.55)	^99^
Hyaloid membrane permeability (× 10^−5^ cm/s)	1.91 (1.24, 3.92)	0.874 (0.616, 1.42)	^99^

Estimated permeability parameters with 95% confidence intervals (where provided) from different sources, all determined fitting models to rabbit data.

### Numerical Methods

All geometries were built using COMSOL Multiphysics.^102^ In constructing each geometry, a mesh for the finite element numerical method was also constructed within COMSOL, with sufficient grid resolution to ensure numerical convergence. This was tested by confirming that further mesh refinement had no impact on example results at the resolution of plotting presented.

The equations were solved and the figures were generated using COMSOL Multiphysics software,^102^ using the implemented stationary solver. Regression lines were obtained with the Scikit-learn library^103^ in Python. The data and the code used to produce the results are available at the GitHub repository: https://github.com/patricia-lamy/MFPT-ocular-drug-delivery.

### Global Sensitivity Analysis

We performed a global sensitivity analysis to identify which geometrical parameters are most important to accurately determine when constructing computational ocular models. We varied the geometrical parameters *a*, *b*, *l*_*D*_, and *l*_*T*_ (see Fig. 1 for their definition) within the range of literature ocular values identified in Table 1 for each species and *h*_*va*_ within ±10% of its base value. We performed this sensitivity analysis for the human, cynomolgus monkey, and rabbit eye models, as the geometries for the rat and mouse models were only well defined for a subset of the parameter combinations. We analyzed the effect of the geometrical parameters on the MFPT for an injection at *P*_*m*_. This was implemented using the eFAST sensitivity method,^104^^,^^105^ with the Python SAlib library,^106^^,^^107^ and with the choice of sampling parameters guided by the methodology proposed in the referenced sources. We set to 4 the number of harmonics to sum in the Fourier series decomposition and to 337 the number of samples to generate. The implementation of the sensitivity analysis sampling was validated by confirming that a dummy variable has sensitivity indices of around zero, demonstrating minimum sampling artifact.^105^ See Supplementary S1.3 for more information.

## Results

### Mean First-Passage Time

**Figure 5.**
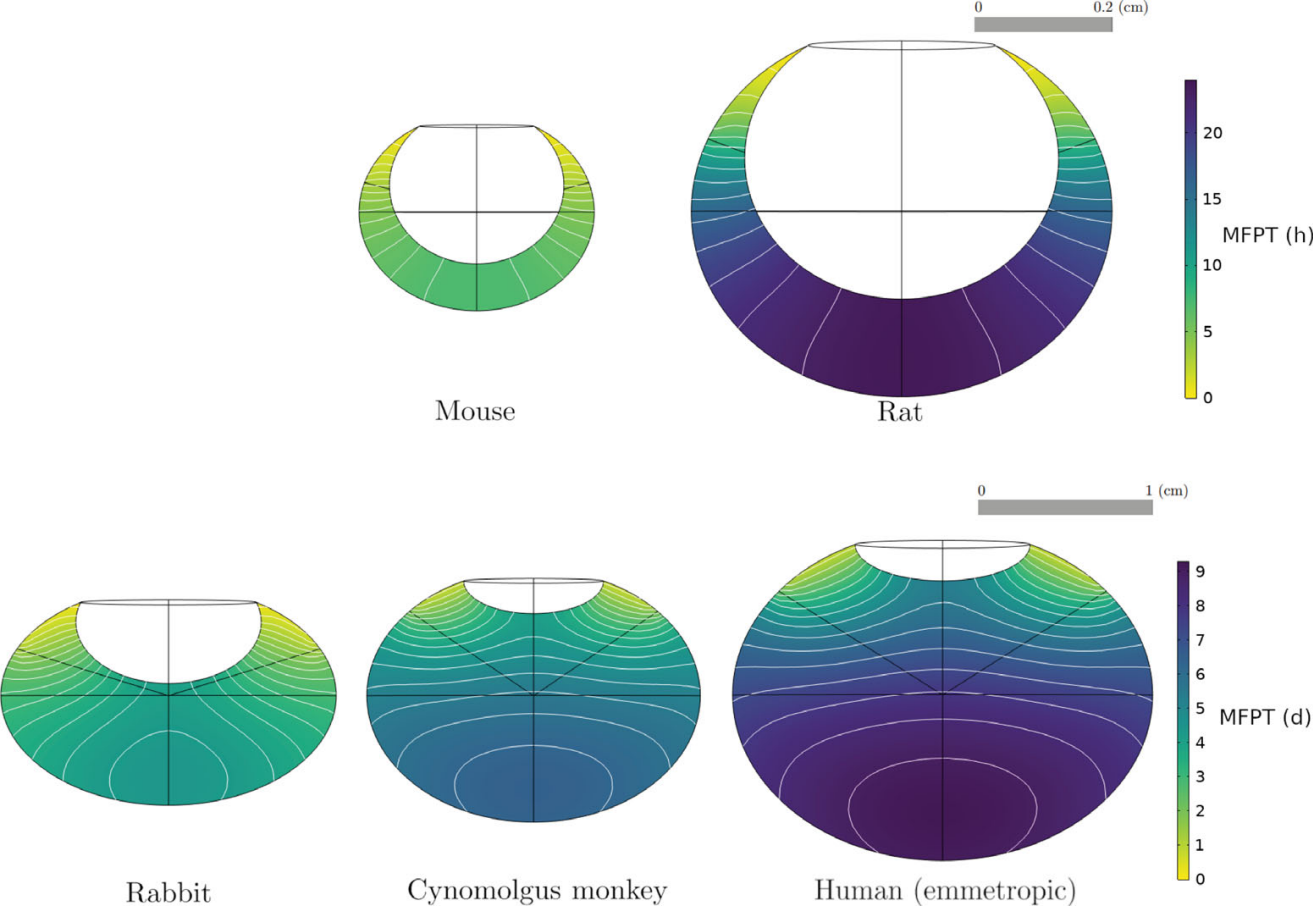
Numerical solution of the mean first-passage time (MFPT) for a Fab molecule in different species, as a function of injection site, with the parameterization of Tables 1 and 2. Contour lines of MFPT are in *white*, while the colormap indicates the MFPT value at any given point in the vitreous chamber. *Black lines* are associated with the construction of the model geometry (Fig. 2).

**Figure 6.**
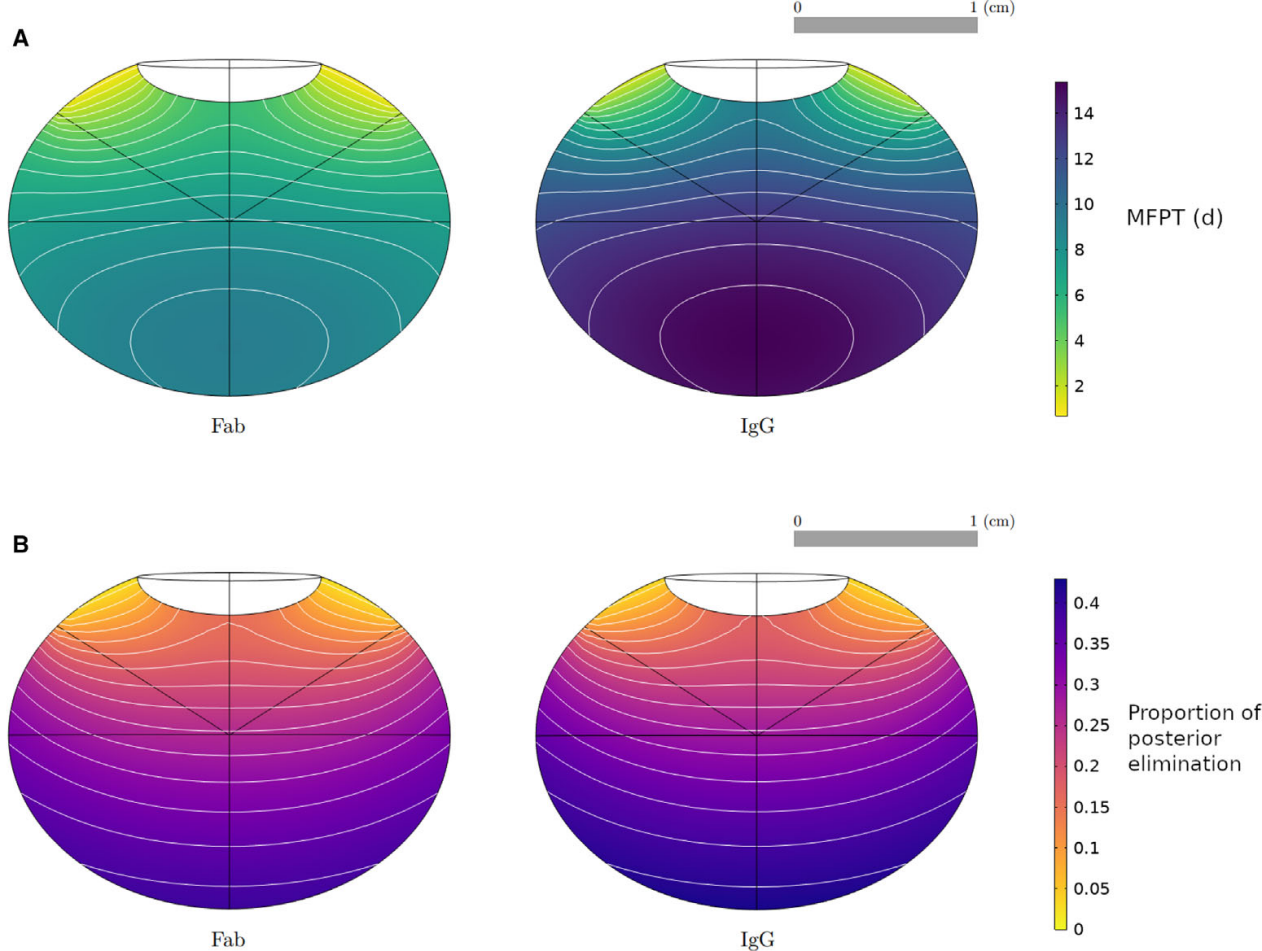
Numerical solution of the MFPT for Fab and IgG molecules in the human emmetropic eye model (**A**) and of the proportion of the drug dose exiting through the vitreous–retina interface (**B**), as a function of injection site, using the parameterization of Tables 1 and 2. The Fab results are also presented with a different color bar scaling in Figures 5 and 8.

To obtain numerical solutions for the MFPT, we solved Equation (1) with parameter values defined in Tables 1 and 2, using the ocular geometries of each species (Fig. 2). The results for the Fab molecule are illustrated in Figure 5. In each model, the MFPT is maximized for an injection site at the back of the vitreous and decreases for injections closer to the aqueous humor. The same behavior was observed for the MFPT of an IgG molecular format, with overall longer residence times. Figure 6A compares the MFPT in the human model for a Fab and IgG molecule, with the maximum MFPT being 9 days and 14 days, respectively.

We observed that the MFPT decreases with eye size, with an MFPT of less than 1 day for all injection sites in the mouse and rat models (Fig. 5). The global sensitivity analysis identified that the length of the semi-axis *b* was the most influential parameter for the MFPT for an injection at *P*_*m*_ (results shown in Supplementary S1.3). The analysis also revealed that the model was sensitive to the permeability parameters when they were varying within their uncertainty range but not when their range varied proportionally with the other parameters (Supplementary S1.3).

**Figure 7.**
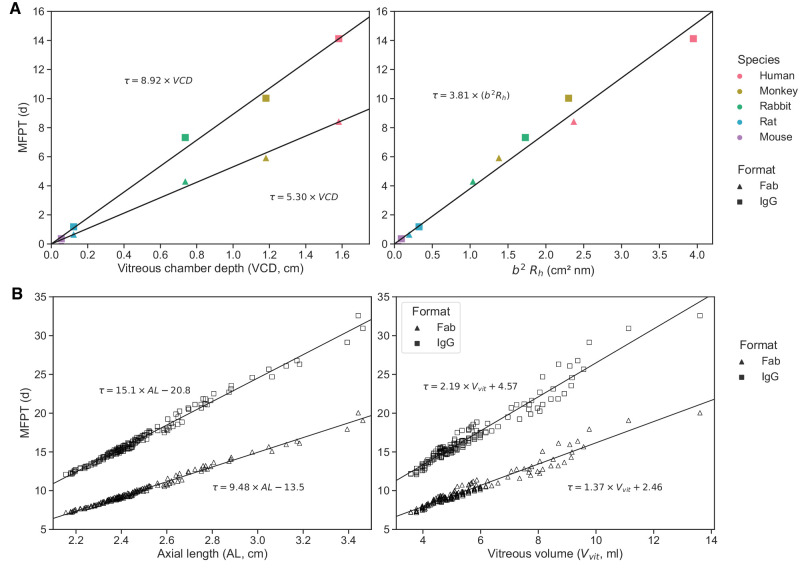
Numerical solution (*symbols*) and linear regressions (*lines*) of the MFPT for different molecular formats injected at *P*_*m*_, using the parameterization of Tables 1 and 2. The MFPT is shown for (**A**) the species-specific geometries of Figure 2 and (**B**) the ensemble of human eye models of Figure 4.

To refine the comparison of the MFPT across species, the MFPT for a Fab and IgG molecular format was solved for an injection at *P*_*m*_ and was plotted against the vitreous chamber depth measure of each species (Fig. 7A). The choice of *P*_*m*_ as a comparison point for the MFPT between species should not significantly influence the results, as the MFPT at a point is a good approximation of the average MFPT of a spherical bolus centered at that point, as long as the radius of the bolus is not too large (see Supplementary S1.4 for more information). The corresponding linear regressions were derived and constrained to go through the origin, as the intercept confidence interval included the origin and as it is the expected theoretical behavior. Following the analysis in Caruso et al. (2020),^8^ where the half-life for each species was plotted as a linear function of r vit 2×Rh with species-specific proportionality constants, the MFPT was also plotted as a function of *b*^2^ × *R*_*h*_. In order to represent the higher level of detail in the eye geometries suggested in this work, the semi-axis *b* was taken to represent the different vitreous sizes (given it was the most sensitive geometrical parameter). The results (shown in Fig. 7A) illustrate proportionality without the need to distinguish between species as all MFPT points are aligned on a linear regression line. A linear relationship for the MFPT across species and molecular formats was obtained, with a regression going through the origin and a slope of 3.81 days/(cm^2^ nm). Equation (2) was used to obtain a slope of 2.64 days/(cm^2^ nm) for *t*_1/2_.

Considering an injection of a Fab or an IgG at *P*_*m*_, Figure 7B shows the MFPT as a function of the AL and of vitreous volume for the ensemble of human eye models. We found that the pathological myopia eyes did not significantly affect the trends found in the ensemble of healthy human eyes, with a change of slope of less than 10% for each molecular format when we excluded the eye geometries constructed using the pathological myopia measurements from Zhou et al.^59^ (results presented in Supplementary S1.5). Comparing the two panels of Figure 7B, we see that the MFPT is better predicted by the linear relation with the AL than with the vitreous volume, with the resulting residence times being more sparsely distributed when plotted against the vitreous volume. As mentioned above, these results are not expected to be sensitive to the chosen injection point *P*_*m*_ and should also hold for larger injection regions (see Supplementary S1.4 for more information).

Using Equation (2), we derived *t*_1/2_ for all species from the MFPT for an injection located at *P*_*m*_. The results are summarized in Table 4, along with the experimental *t*_1/2_ for each species. For the human eye model, we estimated a range of *t*_1/2_ using the MFPT for hypermetropic to myopic eyes illustrated in Figure 7B, hence excluding pathologically myopic eyes, and found this estimated range to be broadly consistent with the spread of *t*_1/2_ observed in normal eyes. For the human, cynomolgus monkey, and rabbit eye models, a range of *t*_1/2_ was also estimated by solving the MFPT within the injection region (identified in Fig. 2). Agreement between simulation and experiment holds for the human and the rabbit, whereas the model's half-lives are overestimated for the cynomolgus monkey and the rat and underestimated for the mouse.

**Table 4.** MFPT and Estimated *t*_1/2_ Modeling Results by Species

		Fab			IgG		
		Modeling Results (Days)		Experimental Results (Days)	Modeling Results (Days)		Experimental Results (Days)
Species		MFPT	*t*_1/2_	*t*_1/2_	MFPT	*t*_1/2_	*t*_1/2_
Mouse	Midpoint *P*_*m*_	0.20	0.14	0.86^38^	0.36	0.25	NA
Rat	Midpoint *P*_*m*_	0.67	0.46	NA	1.18	0.82	0.341^41^^,^^108^
Rabbit	Midpoint *P*_*m*_	4.32	2.99	3.0 (2.75, 3.31)^8^	7.32	5.07	5.4 (4.17, 7.06)^8^
	Injection range	(4.16, 4.39)	(2.88, 3.04)		(7.08, 7.43)	(4.91, 5.15)	
Cynomolgus	Midpoint *P*_*m*_	5.94	4.12	2.4 (2.17, 2.9)^8^	10.03	6.95	3.3 (2.8, 3.90)^8^
monkey	Injection range	(4.94, 6.42)	(3.42, 4.45)		(8.43, 10.76)	(5.84, 7.46)	
Human	Midpoint *P*_*m*_	8.44	5.85	6.5 (5.24, 8.6)^8^	14.12	9.79	9.3 (7.16, 11.67)^8^
(emmetropic)	Injection range	(6.76, 9.22)	(4.69, 6.39)		(11.45, 15.31)	(7.94, 10.61)	
	Ensemble range	(7.52, 10.74)	(5.21, 7.44)		(12.63, 17.79)	(8.75, 12.33)	

MFPT and Estimated *t*_1/2_ (Using Equation (2)) for an Injection at Midpoint *P*_*m*_, for the Injection Range (Injection Location Region Identified in Fig. 2) Reporting the Min-Max Results, and for the Ensemble Range (for an Injection at *P*_*m*_ in the Ensemble of Human Eye Models Excluding the Pathological Myopia Data) Reporting the 5th to 95th Percentile.

The experimental *t*_1/2_ (mean, with lower and upper bound of found interval) for each species and molecular format are also reported.

### Drug Elimination

**Figure 8.**
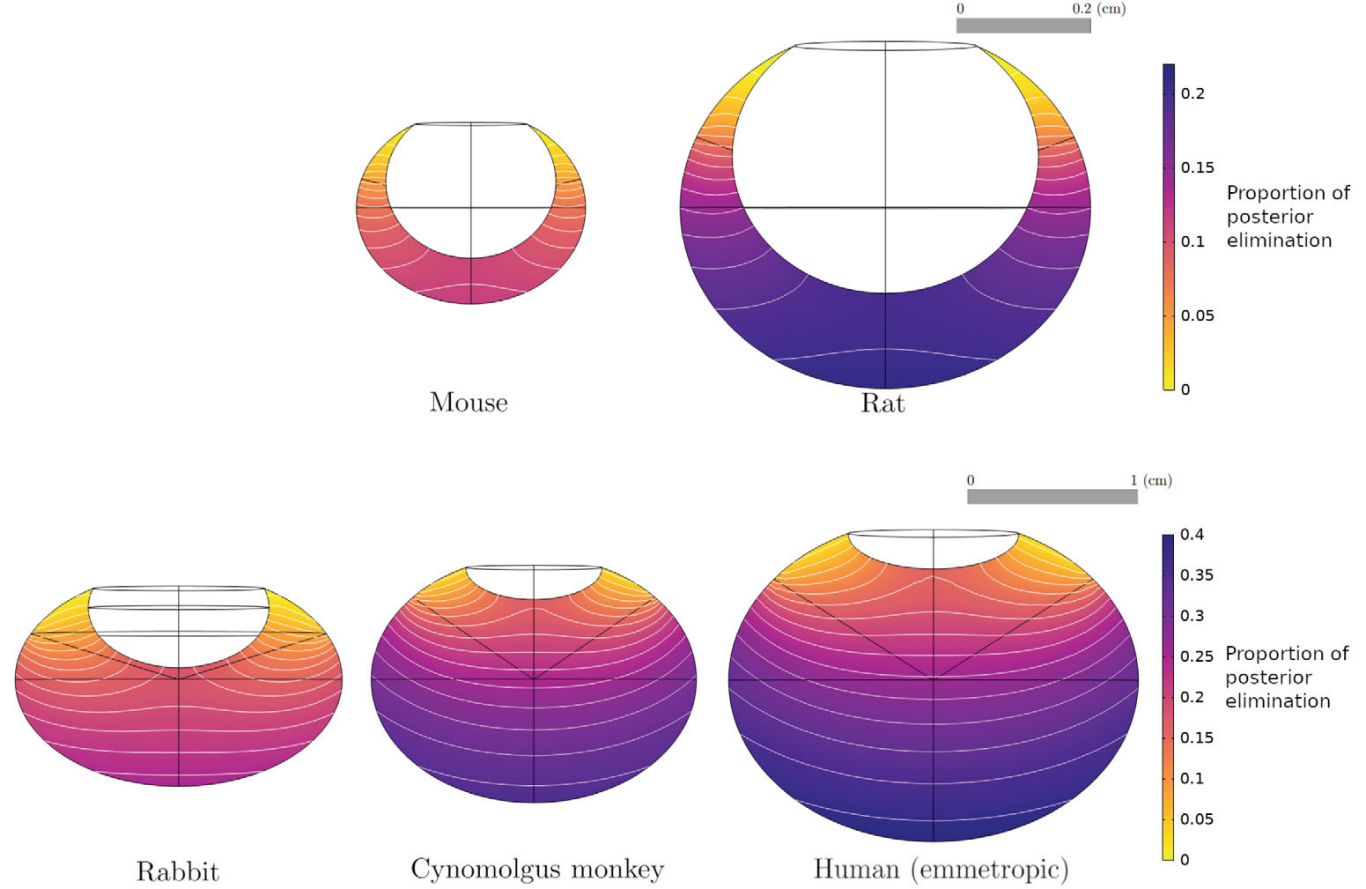
Numerical solution and contour lines of the proportion of drug dose exiting through the vitreous–retina interface (unitless), for a Fab molecule and for each species, as a function of injection site, with the parameterization of Tables 1 and 2, and the geometries in Figure 2.

To obtain the numerical solutions for the proportion of drug exiting through the vitreous–retina interface, Equation (3) was solved with the parameters and ocular geometries of Table 2 and Figure 2, respectively. The results for the Fab molecule are illustrated in Figure 8. The maximum contribution of posterior elimination varied across species, with up to 40% of dose permeating the vitreous–retina interface in the human, versus 12% in the mouse, while the posterior elimination from the injection point *P*_*m*_ varied from 10% to 30% across species. Injection sites located at the back of the eye were associated with higher proportions of posterior elimination, which decreased with the distance from the anterior segment. Similar results were obtained for IgG, as visible in Figure 6B for the emmetropic human eye.

**Figure 9.**
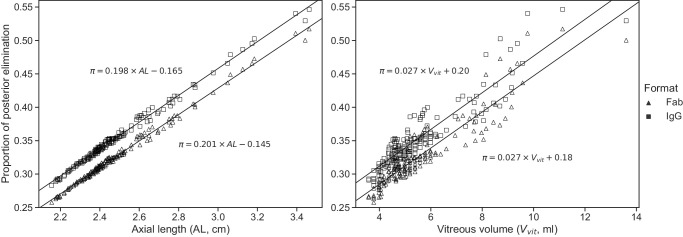
Numerical solution and regression lines of the proportion of drug dose exiting through the vitreous–retina interface in the ensemble of human eye models (as identified in Fig. 4) for an injection at *P*_*m*_ and with parameters of Table 2, as a function of the AL and vitreous volume.

To study the influence of interindividual anatomical differences, the ensemble of human eye models was solved for the posterior elimination after injection at *P*_*m*_ (Fig. 9). The posterior contribution to ocular elimination showed a strong correlation with the AL and vitreous volume for both Fab and IgG, with poor separation between molecular formats for the latter.

### Conditional MFPT

**Figure 10.**
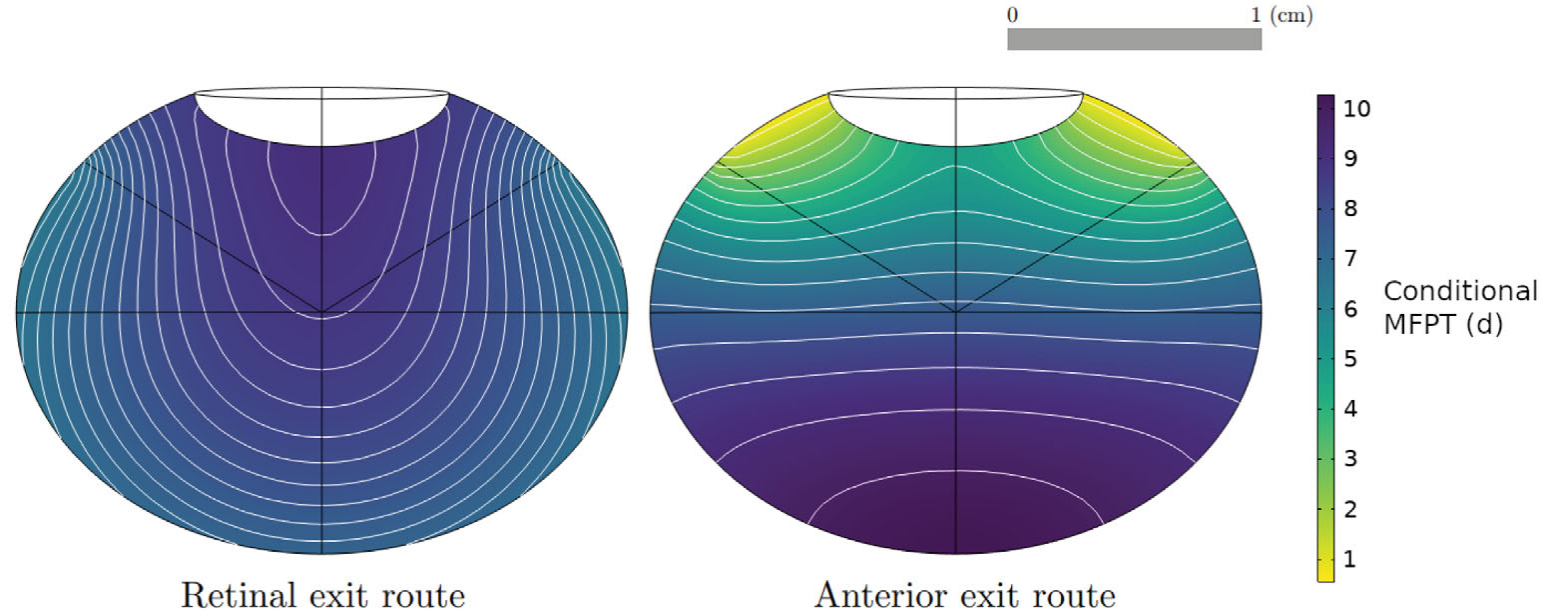
Numerical solution and contour lines for the MFPT, conditional on exiting through the vitreous–retina (*left*) and vitreous–aqueous humor (*right*) interfaces, in the human (emmetropic) eye model for a Fab molecule, as a function of injection site, with the parameterization of Tables 1 and 2.

To obtain numerical solutions for the conditional MFPT, which gives the duration time conditioned on the exit rate, Equations (3) and (4) were solved with parameter values given in Table 2 for a Fab molecule, using the human eye geometry. Figure 10 shows the results for the conditional MFPT. The conditional MFPT for the drug exiting through the vitreous–retina interface has a lower variation range, with exit times varying between 6.5 and 9 days, and has very different contour plots to those of the unconditional MFPT (Figs. 5, 6). In contrast, the conditional MFPT for the drug exiting through the vitreous–aqueous humor interface has a similar range of values and contour plots compared with the unconditional MFPT, indicating that the dynamics in the MFPT solutions could be dominated by the dynamics of the anterior elimination pathway. This behavior was observed in all modeled species (results presented in Supplementary S1.5) and held for the IgG molecular format.

## Discussion

We have given the governing equations for the mean first-passage time, MFPT, the MFPT conditioned on the exit route, and the proportion of drug exiting through subsections of the eye for Fab and IgG molecular formats. We built realistic 3D eye geometries based on ocular measurements and confirmed their anatomical accuracy by comparing them to MRI images, showing that the geometries capture reasonably well the relative size and position of the vitreous chamber and lens (Fig. 3). While the cynomolgus monkey model eccentricity exceeds that of the image, we remark that the image was not obtained in situ and the isolated organ was not subject to the same external forces, that alterations can occur during the tissue processing and fixation, and that all of these causes can affect its appearance. We solved the equations on the ocular geometries, and we analyzed our results to assess the potential influence of spatial parameters on ocular drug residence times. To better study the different geometries, we compared the MFPT at plausible injection points *P*_*m*_, and we showed that neglecting the bolus aspect of the injection has no significant impact on results (Supplementary S1.4). We linked this analysis to ocular half-life and contrasted it with experimental data from nonclinical species and humans.

The MFPT approach modeled the distribution and elimination of Fab and IgG molecules after IVT administration as diffusion-driven processes. While this assumption is supported by previous experiments and modeling work,^53^^–^^57^ the consensus on whether bulk flow plays a significant role for drug distribution in the vitreous is being questioned,^109^ with several authors noting its importance in particular cases.^110^^–^^113^ The approach presented in this work also assumed that no fluid flow was generated during drug injection, a simplification justified by previous modeling work,^114^ though extensions to consider the prospective impact of fluid flow are an interesting future direction for this study.

This study's aims included exploring the importance of injection site location and individual variation for IVT administration of protein therapeutics. The model simulations show that the injection location had a significant effect on the MFPT in all species (Fig. 5). This is in line with a previous computational study, where the influence of four different injection locations in a human eye model was investigated,^115^^,^^116^ concluding that injections at the back of the eye induced higher drug concentrations in the first 24 hours after injection^115^ and maximal drug absorption at the macula.^116^ In the rodent models, the MFPT was higher for injection sites centered on the vitreous chamber depth along the optical axis behind the lens in the vitreous, but these may not be accessible due to the curvature of the lens. We postulate that variations in reported ocular half-lives (both in clinical and nonclinical species) could be partially explained by different injection site locations in the vitreous chamber. In the human eye, the results indicate a large posterior region of the vitreous body where IVT injections are expected to yield maximal drug residence time and retinal permeation (Figs. 5, 6, 8). Conversely, injections closer to the anterior segment lead to lower elimination across the retina. It is expected that alternative ocular delivery approaches to IVT injection, such as implants and sustained-release formulations, similarly ought to target the central and posterior vitreous for improved retinal exposure.

The results of this work agree with previous reports that ocular PK depends on eye and molecular size.^8^^,^^40^ A linear relation was found between the MFPT and the vitreous chamber depth, for both Fab and IgG molecular formats (Fig. 7A). Also, the *t*_1/2_ was linearly correlated to the product of the eye semi-axis *b* squared and the protein *R*_*h*_, with the associated regression line having a slope of 2.64 days/(cm^2^ nm). This is comparable to the range previously reported by Caruso et al.^8^ (1.3–2.4 days/(cm^2^ nm)) for the linear regression of half-life on $r_{vit}^{2}\times R_{h}$ in different species, where *r*_*vit*_ is the vitreal radius. The experimental estimates of that study suggest the relationship is species-specific, with the minipig exhibiting the steepest slope (2.4 days/(cm^2^ nm)) and the rabbit, human, rat, and monkey displaying shallower lines (2.1, 1.8, 1.6, and 1.3 days/(cm^2^ nm), respectively). Interestingly, this sequence of species is not ordered by vitreous size; for example, the vitreal volume in the rabbit is smaller (and thus, diffusion distance is shorter) than both human and monkey vitreous (Table 1). This suggests that factors other than eye size must play a role in determining the PK differences observed across species. Vitreal chamber shape and eccentricity have been proposed as possible determinants,^8^ a notion that is not supported by the present results as the anatomically realistic description of ocular geometries in this work produced the same linear estimates of residence time across all species (Fig. 7A).

The model simulations yielded half-life values aligned with interspecies differences in vitreal volume (i.e., longer half-lives in larger eyes) (Table 4). While the human and rabbit *t*_1/2_ estimates are close to the experimental values, a discrepancy is found for the cynomolgus monkey and for the rodents, although the *t*_1/2_ in the latter is not as well established experimentally as in the larger species. We conjecture that the partial mismatch between experimental data and simulation outcomes can be attributed to the assumptions made regarding the permeability parameters. Notably, including species-specific permeability parameters in the present model can significantly influence the relative contribution of the anterior and posterior pathway to drug elimination. The proportion of drug exiting through the posterior pathway is directly linked to the two permeability parameters within the model. The relative contribution in exit pathways has been previously proposed as a potential determinant of *t*_1/2_ in ocular PK.^8^^,^^40^ Moreover, insights from a prior study on topically applied small molecules indicate variations in ocular tissue permeability across different species.^100^ Extending this understanding to IVT macromolecules, species-specific permeability data may be necessary to obtain more accurate modeling results. While a few studies have previously investigated the effect of molecular size on the permeability of ocular tissues,^29^^,^^117^^,^^118^ there is a lack of comparative studies on the permeability of IVT macromolecules across species.

Our global sensitivity analysis showed that the axial length is the most influential parameter on residence time across species (Supplementary S1.3), confirming the importance of eccentricity in modeling the vitreous chamber. On the other hand, the spherical approximation used in several previous works^41^^,^^119^^–^^122^ implies that the semi-axis *b* has the same length as semi-axis *a*. By way of example, if we compare the injection of a Fab molecule at *P*_*m*_ in the human emmetropic eye model (*a* = 1.1275 cm, *b* = 0.889 cm) and in a spherical model of the same vitreous volume (*a* = *b* = 1.043 cm), we find a meaningful discrepancy in the estimate of the half-life (respectively 5.85 and 7.16 days). Hence, we recommend that future models, aiming to explore PK across various species, do away with the spherical approximation.

The global sensitivity analysis also identified that, considering the high uncertainty on the permeability parameters, the MFPT results were sensitive to the values chosen, although less sensitive than for the values of the axial length. Otherwise, the sensitivity analysis revealed that the model was not particularly sensitive to the permeability parameters when they were varied proportionally with the other parameters (Supplementary S1.3). While the choice of the permeability parameters will affect the modeled clearance times in absolute terms, relative changes such as the proportion of posterior elimination should be insensitive as long as the ratio between the two permeability parameters remains approximately fixed.

Previous work has investigated the impact of eye size on PK in diverse animal species. To the best of our knowledge, this is the first report showing a significant impact of eye shape and eccentricity within the same species, namely, humans. Ocular half-life estimates are known to exhibit sizable differences between patients in clinical studies. For example, Avery et al.^123^ reported a mean *t*_1/2_ of 5.8 days with a standard deviation of 1.8 days for the Fab ranibizumab, and Meyer et al.^124^ reported an average and 95% confidence interval of 11.17 (8.7, 18.2) days following a 3-mg injection of the IgG bevacizumab. On the other hand, a previous study in 41 eyes found no correlation between intraocular drug concentration and AL.^125^ Such contrast of results motivated this research to better understand whether interindividual differences in ocular geometry may impact PK. In the ensemble of human eyes having different volumes and axial lengths, our model showed a large variation in residence time (Fig. 7B), contributing to explain the experimental interindividual variability. The different slopes between the Fab and IgG molecules suggest that the variability in the AL of the eye elongation is more influential on the residence time for molecules with slower diffusion. The linear relation found between MFPT and AL suggests that measuring the AL is sufficient to obtain an estimate of the residence time for any given human eye. This has promising implications for clinical practice, as AL measurements can be obtained in patients with relative ease, more so than the vitreous volume. Optical biometry, both reasonably simple and cost-effective, could serve as a potential stride toward personalized treatment by furnishing insights into individual durability of ocular exposure and pharmacology.

The model also suggests that the proportion of posterior elimination varies greatly between species (Fig. 8), which appears to be mostly correlated to the distance between the injection site and the vitreous–aqueous humor interface. In the rabbit eye model, the posterior pathway contribution to drug elimination was 19% to 23% (Fig. 8) for an injection within the region identified in Figure 2, which is in line with previous experimental estimates. A prior study in rabbit eyes estimated the posterior clearance as 3% to 20% of the dose administered by IVT injection.^78^ Another computational model calculated the percentage of Fab molecules exiting through the RPE to be 12.7% of the IVT dose in a rabbit experiment.^23^ To our knowledge, current estimates are informed by experimental data obtained in rabbits, while Figure 8 highlights clear species differences, making it necessary to further study the elimination pathways in other species. In the ensemble of human eyes, we found that posterior elimination of both Fab and IgG formats is linearly correlated with the axial length and vitreous volume (Fig. 9), strengthening the notion that individual variations in eye shape may influence drug disposition and pharmacology.

The results of the MFPT conditioned on the exit route have provided further information on the dynamics of the MFPT. In all species, the solutions of the conditional MFPT show that the clearance pathway through the vitreous–aqueous humor interface is dominating the behavior in the MFPT solutions. Furthermore, the model suggests that drug molecules leaving through the retina are spending more time in the vitreous chamber than molecules exiting through the aqueous humor (Fig. 10), and the duration time of drug that exits into the target region is approximately 10% longer than the mean duration time. Therefore, while the half-life underestimates the duration of drug in the vitreous that reaches the target, it is still a good measure of duration.

In conclusion, the residence time and the posterior elimination were studied across multiple species used in ocular research and drug development, with the aim to strengthen the interspecies translation of pharmacokinetic and pharmacodynamic studies. The anterior pathway was identified as the predominant route of drug elimination, and the contribution of the posterior pathway is predicted to vary significantly across species. The injection location was found to be highly influential in the drug kinetics, and maximum efficacy was obtained for injections in the posterior vitreous. Additionally, we showed that the variability in vitreous chamber size and shape in human eyes can lead to significant differences in drug residence times and proportion of posterior elimination. The methodology developed in this study emerges as a potent framework for characterizing the vitreal transport dynamics of current ocular therapeutics. By combining our methodology with species-specific measurements of posterior permeabilities, it would be possible to investigate the efficacy of emerging ocular therapeutics.

## Supplementary Material

Supplement 1

Supplement 2

## References

[1] Matsumura S, Kuo AN, Saw SM. An update of eye shape and myopia. *Eye Contact Lens.* 2019; 45(5): 279–285. 30932926 10.1097/ICL.0000000000000571

[2] Atchison DA, Jones CE, Schmid KL, et al. Eye shape in emmetropia and myopia. *Invest Ophthalmol Vis Sci.* 2004; 45(10): 3380–3386. 15452039 10.1167/iovs.04-0292

[3] Strang NC, Schmid KL, Carney LG. Hyperopia is predominantly axial in nature. *Curr Eye Res.* 1998; 17(4): 380–383. 9561829 10.1080/02713689808951218

[4] Quillen DA . Common causes of vision loss in elderly patients. *Am Fam Physician.* 1999; 60(1): 99–108. 10414631

[5] Mitchell P, Liew G, Gopinath B, Wong TY. Age-related macular degeneration. *Lancet.* 2018; 392(10153): 1147–1159. 30303083 10.1016/S0140-6736(18)31550-2

[6] Chappelow AV, Kaiser PK. Neovascular age-related macular degeneration. *Drugs.* 2008; 68(8): 1029–1036. 18484796 10.2165/00003495-200868080-00002

[7] Shweiki D, Itin A, Soffer D, Keshet E. Vascular endothelial growth factor induced by hypoxia may mediate hypoxia-initiated angiogenesis. *Nature.* 1992; 359(6398): 843–845. 1279431 10.1038/359843a0

[8] Caruso A, Füth M, Alvarez-Sánchez R, et al. Ocular half-life of intravitreal biologics in humans and other species: meta-analysis and model-based prediction. *Mol Pharmaceutics.* 2020; 17(2): 695–709. 10.1021/acs.molpharmaceut.9b01191 31876425

[9] Shatz W, Hass PE, Mathieu M, et al. Contribution of antibody hydrodynamic size to vitreal clearance revealed through rabbit studies using a species-matched Fab. *Mol Pharmaceutics.* 2016; 13(9): 2996–3003. 10.1021/acs.molpharmaceut.6b00345 27244474

[10] Meyer C, Holz F. Preclinical aspects of anti-VEGF agents for the treatment of wet AMD: ranibizumab and bevacizumab. *Eye.* 2011; 25(6): 661–672. 21455242 10.1038/eye.2011.66 PMC3178135

[11] Rosenfeld PJ, Brown DM, Heier JS, et al. Ranibizumab for neovascular age-related macular degeneration. *N Engl J Med.* 2006; 355(14): 1419–1431. 17021318 10.1056/NEJMoa054481

[12] Brown DM, Kaiser PK, Michels M, et al. Ranibizumab versus verteporfin for neovascular age-related macular degeneration. *N Engl J Med.* 2006; 355(14): 1432–1444. 17021319 10.1056/NEJMoa062655

[13] Bernoff AJ, Lindsay AE, Schmidt DD. Boundary homogenization and capture time distributions of semipermeable membranes with periodic patterns of reactive sites. *Multiscale Model Simul.* 2018; 16(3): 1411–1447.

[14] Krohne TU, Eter N, Holz FG, Meyer CH. Intraocular pharmacokinetics of bevacizumab after a single intravitreal injection in humans. *Am J Ophthalmol.* 2008; 146(4): 508–512. 18635152 10.1016/j.ajo.2008.05.036

[15] Krohne TU, Liu Z, Holz FG, Meyer CH. Intraocular pharmacokinetics of ranibizumab following a single intravitreal injection in humans. *Am J Ophthalmol.* 2012; 154(4): 682–686. 22818800 10.1016/j.ajo.2012.03.047

[16] Xing L, Dorrepaal SJ, Gale J. Survey of intravitreal injection techniques and treatment protocols among retina specialists in Canada. *Can J Ophthalmol.* 2014; 49(3): 261–266. 24862772 10.1016/j.jcjo.2014.03.009

[17] Aiello LP, Brucker AJ, Chang S, et al. Evolving guidelines for intravitreous injections. *Retina.* 2004; 24(5): S3–S19. 15483476 10.1097/00006982-200410001-00002

[18] Peyman GA, Lad EM, Moshfeghi DM. Intravitreal injection of therapeutic agents. *Retina.* 2009; 29(7): 875–912. 19584648 10.1097/IAE.0b013e3181a94f01

[19] Fagan XJ, Al-Qureshi S. Intravitreal injections: a review of the evidence for best practice. *Clin Exp Ophthalmol.* 2013; 41(5): 500–507. 23078366 10.1111/ceo.12026

[20] Chong V . Ranibizumab for the treatment of wet AMD: a summary of real-world studies. *Eye.* 2016; 30(2): 270–286. 26634711 10.1038/eye.2015.217 PMC4763117

[21] Doshi RR, Bakri SJ, Fung AE. Intravitreal injection technique. *Semin Ophthalmol*. 2011; 26: 104–113. 21609222 10.3109/08820538.2010.541318

[22] Del Amo EM, Rimpelä AK, Heikkinen E, et al. Pharmacokinetic aspects of retinal drug delivery. *Prog Retin Eye Res.* 2017; 57: 134–185. 28028001 10.1016/j.preteyeres.2016.12.001

[23] Hutton-Smith LA, Gaffney EA, Byrne HM, Maini PK, Gadkar K, Mazer NA. Ocular pharmacokinetics of therapeutic antibodies given by intravitreal injection: estimation of retinal permeabilities using a 3-compartment semi-mechanistic model. *Mol Pharmaceutics.* 2017; 14(8): 2690–2696. 10.1021/acs.molpharmaceut.7b00164 28631484

[24] Ameri H, Chader GJ, Kim JG, Sadda SR, Rao NA, Humayun MS. The effects of intravitreous bevacizumab on retinal neovascular membrane and normal capillaries in rabbits. *Invest Ophthalmol Vis Sci.* 2007; 48(12): 5708–5715. 18055823 10.1167/iovs.07-0731

[25] Ahn SJ, Ahn J, Park S, et al. Intraocular pharmacokinetics of ranibizumab in vitrectomized versus nonvitrectomized eyes. *Invest Ophthalmol Vis Sci.* 2014; 55(1): 567–573. 24398088 10.1167/iovs.13-13054

[26] Gaudreault J, Fei D, Beyer JC, et al. Pharmacokinetics and retinal distribution of ranibizumab, a humanized antibody fragment directed against VEGF-A, following intravitreal administration in rabbits. *Retina.* 2007; 27(9): 1260–1266. 18046235 10.1097/IAE.0b013e318134eecd

[27] Bakri SJ, Snyder MR, Reid JM, Pulido JS, Ezzat MK, Singh RJ. Pharmacokinetics of intravitreal ranibizumab (Lucentis). *Ophthalmology.* 2007; 114(12): 2179–2182. 18054637 10.1016/j.ophtha.2007.09.012

[28] Christoforidis JB, Carlton MM, Knopp MV, Hinkle GH. PET/CT imaging of I-124–radiolabeled bevacizumab and ranibizumab after intravitreal injection in a rabbit model. *Invest Ophthalmol Vis Sci.* 2011; 52(8): 5899–5903. 21685343 10.1167/iovs.10-6862

[29] Kim HM, Han H, Hong HK, et al. Permeability of the retina and RPE-choroid-sclera to three ophthalmic drugs and the associated factors. *Pharmaceutics.* 2021; 13(5): 655. 34064405 10.3390/pharmaceutics13050655 PMC8147773

[30] Niwa Y, Kakinoki M, Sawada T, Wang X, Ohji M. Ranibizumab and aflibercept: intraocular pharmacokinetics and their effects on aqueous VEGF level in vitrectomized and nonvitrectomized macaque eyes. *Invest Ophthalmol Vis Sci.* 2015; 56(11): 6501–6505. 26447985 10.1167/iovs.15-17279

[31] Gaudreault J, Fei D, Rusit J, Suboc P, Shiu V. Preclinical pharmacokinetics of Ranibizumab (rhuFabV2) after a single intravitreal administration. *Invest Ophthalmol Vis Sci.* 2005; 46(2): 726–733. 15671306 10.1167/iovs.04-0601

[32] Miyake T, Sawada O, Kakinoki M, et al. Pharmacokinetics of bevacizumab and its effect on vascular endothelial growth factor after intravitreal injection of bevacizumab in macaque eyes. *Invest Ophthalmol Vis Sci.* 2010; 51(3): 1606–1608. 19875666 10.1167/iovs.09-4140

[33] Lu F, Adelman RA. Are intravitreal bevacizumab and ranibizumab effective in a rat model of choroidal neovascularization? *Graefes Arch Clin Exp Ophthalmol.* 2009; 247: 171–177. 18781316 10.1007/s00417-008-0936-y

[34] Gal-Or O, Dotan A, Dachbash M, et al. Bevacizumab clearance through the iridocorneal angle following intravitreal injection in a rat model. *Exp Eye Res.* 2016; 145: 412–416. 26923799 10.1016/j.exer.2016.02.006

[35] Stricker-Krongrad A, Shoemake CR, Bouchard GF. The miniature swine as a model in experimental and translational medicine. *Toxicol Pathol.* 2016; 44(4): 612–623. 27073085 10.1177/0192623316641784

[36] Shrader SM, Greentree WF. Göttingen minipigs in ocular research. *Toxicol Pathol.* 2018; 46(4): 403–407. 29683084 10.1177/0192623318770379

[37] Kelley RF, Tesar DB, Wang Y, et al. Generation of a porcine antibody fab fragment using protein engineering to facilitate the evaluation of ocular sustained delivery technology. *Mol Pharmaceutics.* 2022; 19(5): 1540–1547. 10.1021/acs.molpharmaceut.2c00048 35393854

[38] Bussing D, Li Y, Guo L, Verma A, Sullivan JM, Shah DK. Pharmacokinetics of monoclonal antibody and antibody fragments in the mouse eye following intravitreal administration. *J Pharm Sci.* 2023; 112(8): 2276–2284. 37062415 10.1016/j.xphs.2023.04.006

[39] Schlichtenbrede FC, Mittmann W, Rensch F, Vom Hagen F, Jonas JB, Euler T. Toxicity assessment of intravitreal triamcinolone and bevacizumab in a retinal explant mouse model using two-photon microscopy. *Invest Ophthalmol Vis Sci.* 2009; 50(12): 5880–5887. 19578025 10.1167/iovs.08-3078

[40] Crowell SR, Wang K, Famili A, et al. Influence of charge, hydrophobicity, and size on vitreous pharmacokinetics of large molecules. *Transl Vis Sci Technol.* 2019; 8(6): 1. 10.1167/tvst.8.6.1 PMC6827426 31695962

[41] Hutton-Smith LA, Gaffney EA, Byrne HM, Maini PK, Schwab D, Mazer NA. A mechanistic model of the intravitreal pharmacokinetics of large molecules and the pharmacodynamic suppression of ocular vascular endothelial growth factor levels by ranibizumab in patients with neovascular age-related macular degeneration. *Mol Pharmaceutics.* 2016; 13(9): 2941–2950. 10.1021/acs.molpharmaceut.5b00849 26726925

[42] Missel PJ . Simulating intravitreal injections in anatomically accurate models for rabbit, monkey, and human eyes. *Pharm Res.* 2012; 29(12): 3251–3272. 22752935 10.1007/s11095-012-0721-9 PMC3497967

[43] Lamminsalo M, Taskinen E, Karvinen T, et al. Extended pharmacokinetic model of the rabbit eye for Intravitreal and Intracameral injections of macromolecules: quantitative analysis of anterior and posterior elimination pathways. *Pharm Res.* 2018; 35(8): 1–14. 10.1007/s11095-018-2435-0 29855726

[44] Lamminsalo M, Karvinen T, Subrizi A, Urtti A, Ranta VP. Extended pharmacokinetic model of the intravitreal injections of macromolecules in rabbits. Part 2: parameter estimation based on concentration dynamics in the vitreous, retina, and aqueous humor. *Pharm Res.* 2020; 37(11): 1–14. 10.1007/s11095-020-02946-1 PMC7581578 33094404

[45] Berg HC, Purcell EM. Physics of chemoreception. *Biophys J.* 1977; 20(2): 193–219. 911982 10.1016/S0006-3495(77)85544-6 PMC1473391

[46] Bénichou O, Voituriez R. From first-passage times of random walks in confinement to geometry-controlled kinetics. *Phys Rep.* 2014; 539(4): 225–284.

[47] Redner S . *A Guide to First-Passage Processes*. Cambridge, UK: Cambridge University Press; 2001.

[48] Gardiner C . *Stochastic Methods*. 4th ed. Berlin: Springer; 2009.

[49] Bressloff PC, Newby JM. Stochastic models of intracellular transport. *Rev Mod Phys.* 2013; 85(1): 135–191.

[50] McKenzie HW, Lewis MA, Merrill EH. First passage time analysis of animal movement and insights into the functional response. *Bull Math Biol.* 2009; 71(1): 107–129. 18825463 10.1007/s11538-008-9354-x

[51] Holcman D, Schuss Z. Escape through a small opening: receptor trafficking in a synaptic membrane. *J Stat Phys.* 2004; 117(5): 975–1014.

[52] Newby JM, Seim I, Lysy M, et al. Technological strategies to estimate and control diffusive passage times through the mucus barrier in mucosal drug delivery. *Adv Drug Deliv Rev.* 2018; 124: 64–81. 29246855 10.1016/j.addr.2017.12.002 PMC5809312

[53] Maurice DM . The exchange of sodium between the vitreous body and the blood and aqueous humor. *J Physiol.* 1957; 137(1): 110. 13439588 10.1113/jphysiol.1957.sp005800 PMC1363002

[54] Moseley H, Foulds W, Allan D, Kyle P. Routes of clearance of radioactive water from the rabbit vitreous. *Br J Ophthalmol.* 1984; 68(3): 145–151. 6696868 10.1136/bjo.68.3.145 PMC1040278

[55] Gaul GR, Brubaker RF. Measurement of aqueous flow in rabbits with corneal and vitreous depots of fluorescent dye. *Invest Ophthalmol Vis Sci.* 1986; 27(9): 1331–1335. 2427473

[56] Maurice DM . Flow of water between aqueous and vitreous compartments in the rabbit eye. *Am J Physiol Renal Physiol.* 1987; 252(1): F104–F108. 10.1152/ajprenal.1987.252.1.F104 3812693

[57] Araie M, Maurice D. The loss of fluorescein, fluorescein glucuronide and fluorescein isothiocyanate dextran from the vitreous by the anterior and retinal pathways. *Exp Eye Res.* 1991; 52(1): 27–39. 1714398 10.1016/0014-4835(91)90125-x

[58] Karlin S, Taylor HE. *A Second Course in Stochastic Processes*. New-York: Elsevier; 1981.

[59] Zhou J, Tu Y, Chen Q, Wei W. Quantitative analysis with volume rendering of pathological myopic eyes by high-resolution three-dimensional magnetic resonance imaging. *Medicine.* 2020; 99(42): 1–5. 10.1097/MD.0000000000022685 PMC7572025 33080714

[60] Santana d JM, Cordeiro GG, Soares DTC, Costa MR, Costa Pinto PdA, Lira RPC. Use of axial length to estimate the vitreous chamber volume in pseudophakic. *Graefes Arch Clin Exp Ophthalmol.* 2021; 259: 1471–1475. 33141255 10.1007/s00417-020-04991-3

[61] Azhdam AM, Goldberg RA, Ugradar S. In vivo measurement of the human vitreous chamber volume using computed tomography imaging of 100 eyes. *Transl Vis Sci Technol.* 2020; 9(1): 2. 10.1167/tvst.9.1.2 PMC7255624 32509437

[62] Morgan IG, Ohno-Matsui K, Saw SM. Myopia. *Lancet.* 2012; 379(9827): 1739–1748. 22559900 10.1016/S0140-6736(12)60272-4

[63] Tkatchenko TV, Shen Y, Tkatchenko AV. Analysis of postnatal eye development in the mouse with high-resolution small animal magnetic resonance imaging. *Invest Ophthalmol Vis Sci.* 2010; 51(1): 21–27. 19661239 10.1167/iovs.08-2767 PMC2828876

[64] Hughes A . A schematic eye for the rat. *Vis Res.* 1979; 19(5): 569–588. 483586 10.1016/0042-6989(79)90143-3

[65] Sawada T, Nakamura J, Nishida Y, Kani K, Morikawa S, Inubushi T. Magnetic resonance imaging studies of the volume of the rabbit eye with intravenous mannitol. *Curr Eye Res.* 2002; 25(3): 173–177. 12607187 10.1076/ceyr.25.3.173.13474

[66] Schmucker C, Schaeffel F. In vivo biometry in the mouse eye with low coherence interferometry. *Vis Res.* 2004; 44(21): 2445–2456. 15358080 10.1016/j.visres.2004.05.018

[67] Liu J, Farid H. Twenty-four-hour change in axial length in the rabbit eye. *Invest Ophthalmol Vis Sci.* 1998; 39(13): 2796–2799. 9856794

[68] Pan X, Muir ER, Sellitto C, et al. Age-dependent changes in the water content and optical power of the in vivo mouse lens revealed by multi-parametric MRI and optical modeling. *Invest Ophthalmol Vis Sci.* 2023; 64(4): 24. 10.1167/iovs.64.4.24 PMC10132318 37079314

[69] Massof RW, Chang FW. A revision of the rat schematic eye. *Vis Res.* 1972; 12(5): 793–796. 5037702 10.1016/0042-6989(72)90005-3

[70] Pe’er J, Muckarem M, Zajicek G. Epithelial cell migration in the normal rat lens. *Ann Anat.* 1996; 178(5): 433–436. 8931854 10.1016/S0940-9602(96)80133-6

[71] Werner L, Chew J, Mamalis N. Experimental evaluation of ophthalmic devices and solutions using rabbit models. *Vet Ophthalmol.* 2006; 9(5): 281–291. 16939455 10.1111/j.1463-5224.2006.00495.x

[72] Lozano DC, Twa MD. Development of a rat schematic eye from in vivo biometry and the correction of lateral magnification in SD-OCT imaging. *Invest Ophthalmol Vis Sci.* 2013; 54(9): 6446–6455. 23989191 10.1167/iovs.13-12575 PMC3787660

[73] Atsumi I, Kurata M, Sakaki H. Comparative study on ocular anatomical features among rabbits, beagle dogs and cynomolgus monkeys. *Anim Eye Res.* 2013; 32: 35–41.

[74] Kaplan H, Chiang CW, Chen J, Song SK. Vitreous volume of the mouse measured by quantitative high-resolution MRI. *Invest Ophthalmol Vis Sci.* 2010; 51(13): 4414.

[75] Clough J, Parikh C, Edelhauser H. Anterior chamber, lens and globe volumes in Balb/C and C57/BL6 mice. *Invest Ophthalmol Vis Sci.* 2003; 44(13): 648.

[76] Lin CH, Sun YJ, Lee SH, et al. A protocol to inject ocular drug implants into mouse eyes. *STAR Protocols.* 2022; 3(1): 101143. 35141566 10.1016/j.xpro.2022.101143 PMC8810562

[77] Sha O, Kwong W. Postnatal developmental changes of vitreous and lens volumes in Sprague-Dawley rats. *Neuroembryol Aging.* 2006; 4(4): 183–188.

[78] Del Amo EM, Urtti A. Rabbit as an animal model for intravitreal pharmacokinetics: clinical predictability and quality of the published data. *Exp Eye Res.* 2015; 137: 111–124. 25975234 10.1016/j.exer.2015.05.003

[79] Vézina M . Comparative ocular anatomy in commonly used laboratory animals. In: Weir AB, Collins M, eds. *Assessing Ocular Toxicology in Laboratory Animals*. New-York: Springer; 2013: 1–21.

[80] Jeon CJ, Strettoi E, Masland RH. The major cell populations of the mouse retina. *J Neurosci.* 1998; 18(21): 8936–8946. 9786999 10.1523/JNEUROSCI.18-21-08936.1998 PMC6793518

[81] Lyubarsky AL, Daniele LL, Pugh Jr EN. From candelas to photoisomerizations in the mouse eye by rhodopsin bleaching in situ and the light-rearing dependence of the major components of the mouse ERG. *Vis Res.* 2004; 44(28): 3235–3251. 15535992 10.1016/j.visres.2004.09.019

[82] Drager U, Olsen J. Ganglion-cell distribution in the retina of the mouse. *Invest Ophthalmol Vis Sci.* 1981; 20(3): 285–293. 6162818

[83] Mayhew T, Astle D. Photoreceptor number and outer segment disk membrane surface area in the retina of the rat: stereological data for whole organ and average photoreceptor cell. *J Neurocytol.* 1997; 26(1): 53–61. 9154529 10.1023/a:1018563409196

[84] Baden T, Euler T, Berens P. Understanding the retinal basis of vision across species. *Nat Rev Neurosci.* 2020; 21(1): 5–20. 31780820 10.1038/s41583-019-0242-1

[85] Reichenbach A, Schnitzer J, Friedrich A, Ziegert W, Brückner G, Schober W. Development of the rabbit retina: I. Size of eye and retina, and postnatal cell proliferation. *Anat Embryol.* 1991; 183: 287–297. 10.1007/BF00192216 2042753

[86] Choi KE, Anh VTQ, Yun C, et al. Normative data of ocular biometry, optical coherence tomography, and electrophysiology conducted for cynomolgus macaque monkeys. *Transl Vis Sci Technol.* 2021; 10(13): 14. 10.1167/tvst.10.13.14 PMC8590181 34757392

[87] Lapuerta P, Schein SJ. A four-surface schematic eye of macaque monkey obtained by an optical method. *Vis Res.* 1995; 35(16): 2245–2254. 7571461 10.1016/0042-6989(94)00320-l

[88] Koretz JF, Strenk SA, Strenk LM, Semmlow JL. Scheimpflug and high-resolution magnetic resonance imaging of the anterior segment: a comparative study. *J Opt Soc Am A Opt Image Sci Vis.* 2004; 21(3): 346–354. 15005398 10.1364/josaa.21.000346

[89] Manns F, Parel JM, Denham D, et al. Optomechanical response of human and monkey lenses in a lens stretcher. *Invest Ophthalmol Vis Sci.* 2007; 48(7): 3260–3268. 17591897 10.1167/iovs.06-1376 PMC3429371

[90] Rosen AM, Denham DB, Fernandez V, et al. In vitro dimensions and curvatures of human lenses. *Vis Res.* 2006; 46(6-7): 1002–1009. 16321421 10.1016/j.visres.2005.10.019

[91] Wikler KC, Williams RW, Rakic P. Photoreceptor mosaic: number and distribution of rods and cones in the rhesus monkey retina. *J Comp Neurol.* 1990; 297(4): 499–508. 2384610 10.1002/cne.902970404

[92] Panda-Jonas S, Jonas JB, Jakobczyk M, Schneider U. Retinal photoreceptor count, retinal surface area, and optic disc size in normal human eyes. *Ophthalmology.* 1994; 101(3): 519–523. 8127572 10.1016/s0161-6420(94)31305-4

[93] Curcio CA, Allen KA. Topography of ganglion cells in human retina. *J Comp Neurol.* 1990; 300(1): 5–25. 2229487 10.1002/cne.903000103

[94] Nagra M, Gilmartin B, Thai NJ, Logan NS. Determination of retinal surface area. *J Anat.* 2017; 231(3): 319–324. 28620965 10.1111/joa.12641 PMC5554828

[95] Chui TY, Bissig D, Berkowitz BA, Akula JD. Refractive development in the “ROP rat”. *J Ophthalmol.* 2012; 2012: 1–15. 10.1155/2012/956705 PMC3307090 22482037

[96] Short BG . Safety evaluation of ocular drug delivery formulations: techniques and practical considerations. *Toxicol Pathol.* 2008; 36(1): 49–62. 18337221 10.1177/0192623307310955

[97] Berg HC . *Random Walks in Biology*. Expanded ed. Princeton, NJ: Princeton University Press; 1993.

[98] Delgado MI, Ward MJ, Coombs D. Conditional mean first passage times to small traps in a 3-D domain with a sticky boundary: applications to T cell searching behavior in lymph nodes. *Multiscale Model Simul.* 2015; 13(4): 1224–1258.

[99] Hutton-Smith L . *Modeling the Pharmacokinetics and Pharmacodynamics of Macromolecules for the Treatment of Wet AMD*. PhD thesis. Oxford, UK: University of Oxford; 2018.

[100] Loch C, Zakelj S, Kristl A, et al. Determination of permeability coefficients of ophthalmic drugs through different layers of porcine, rabbit and bovine eyes. *Eur J Pharm Sci.* 2012; 47(1): 131–138. 22659372 10.1016/j.ejps.2012.05.007

[101] Hutton-Smith LA, Gaffney EA, Byrne HM, Caruso A, Maini PK, Mazer NA. Theoretical insights into the retinal dynamics of vascular endothelial growth factor in patients treated with ranibizumab, based on an ocular pharmacokinetic/pharmacodynamic model. *Mol Pharmaceutics.* 2018; 15(7): 2770–2784. 10.1021/acs.molpharmaceut.8b00280 29734810

[102] COMSOL Multiphysics v. 6.2, www.comsol.com. COMSOL AB, Stockholm, Sweden.

[103] Pedregosa F, Varoquaux G, Gramfort A, et al. Scikit-learn: machine learning in Python. *J Mach Learn Res.* 2011; 12: 2825–2830.

[104] Saltelli A, Tarantola S, Chan KS. A quantitative model-independent method for global sensitivity analysis of model output. *Technometrics.* 1999; 41(1): 39–56.

[105] Marino S, Hogue IB, Ray CJ, Kirschner DE. A methodology for performing global uncertainty and sensitivity analysis in systems biology. *J Theor Biol.* 2008; 254(1): 178–196. 18572196 10.1016/j.jtbi.2008.04.011 PMC2570191

[106] Iwanaga T, Usher W, Herman J. Toward SALib 2.0: advancing the accessibility and interpretability of global sensitivity analyses. *Socio-environ Syst Model.* 2022; 4: 18155

[107] Herman J, Usher W. SALib: an open-source Python library for sensitivity analysis. *J Open Source Softw.* 2017; 2(9): 1–2, doi:10.21105/joss.00097.

[108] Chuang LH, Wu WC, Yeung L, et al. Serum concentration of bevacizumab after intravitreal injection in experimental branch retinal vein occlusion. *Ophthalmic Res.* 2010; 45(1): 31–35. 20714188 10.1159/000315617

[109] Wilson CG, Tan LE, Mains J. Principles of retinal drug delivery from within the vitreous. In: Kompella UB, Edelhauser HF, eds. *Drug Product Development for the Back of the Eye*. New-York: Springer; 2011: 125–158.

[110] Xu J, Heys JJ, Barocas VH, Randolph TW. Permeability and diffusion in vitreous humor: implications for drug delivery. *Pharm Res.* 2000; 17: 664–669. 10955838 10.1023/a:1007517912927

[111] Missel PJ . Hydraulic flow and vascular clearance influences on intravitreal drug delivery. *Pharm Res.* 2002; 19: 1636–1647. 12458669 10.1023/a:1020940927675

[112] Park J, Bungay PM, Lutz RJ, et al. Evaluation of coupled convective-diffusive transport of drugs administered by intravitreal injection and controlled release implant. *J Control Release.* 2005; 105(3): 279–295. 15896868 10.1016/j.jconrel.2005.03.010

[113] Smith DW, Lee CJ, Gardiner BS. No flow through the vitreous humor: how strong is the evidence? *Prog Retin Eye Res.* 2020; 78: 100845. 10.1016/j.preteyeres.2020.100845 32035123

[114] Ruffini A, Casalucci A, Cara C, Ethier CR, Repetto R. Drug distribution after intravitreal injection: a mathematical model. *Invest Ophthalmol Vis Sci.* 2024; 65(4): 9. 10.1167/iovs.65.4.9 PMC10996986 38568619

[115] Friedrich S, Cheng YL, Saville B. Drug distribution in the vitreous humor of the human eye: the effects of intravitreal injection position and volume. *Curr Eye Res.* 1997; 16(7): 663–669. 9222083 10.1076/ceyr.16.7.663.5061

[116] Friedmann E, Dörsam S, Auffarth GU. Models and algorithms for the refinement of therapeutic approaches for retinal diseases. *Diagnostics.* 2023; 13(5): 975. 36900119 10.3390/diagnostics13050975 PMC10001150

[117] Yoshihara N, Terasaki H, Shirasawa M, et al. Permeability and anti–vascular endothelial growth factor effects of bevacizumab, ranibizumab, and aflibercept in polarized retinal pigment epithelial layer in vitro. *Retina.* 2017; 37(1): 179–190. 28005721 10.1097/IAE.0000000000001117

[118] Ramsay E, Hagström M, Vellonen KS, et al. Role of retinal pigment epithelium permeability in drug transfer between posterior eye segment and systemic blood circulation. *Eur J Pharm Biopharm.* 2019; 143: 18–23. 31419586 10.1016/j.ejpb.2019.08.008

[119] Jooybar E, Abdekhodaie MJ, Farhadi F, Cheng YL. Computational modeling of drug distribution in the posterior segment of the eye: effects of device variables and positions. *Math Biosci.* 2014; 255: 11–20. 24946303 10.1016/j.mbs.2014.06.008

[120] Dosmar E, Vuotto G, Su X, et al. Compartmental and COMSOL multiphysics 3D modeling of drug diffusion to the vitreous following the administration of a sustained-release drug delivery system. *Pharmaceutics.* 2021; 13(11): 1862. 34834276 10.3390/pharmaceutics13111862 PMC8624029

[121] Khoobyar A, Naghdloo A, Penkova AN, Humayun MS, Sadhal SS. Analytical and computational modeling of sustained-release drug implants in the vitreous humor. *J Heat Transf.* 2021; 143(10): 101201. 10.1115/1.4051785 PMC8597555 35832287

[122] Li H, Zhu X, Wang M, Zhao D, Li H, Yan J. Drug sustained release from degradable drug-loaded in-situ hydrogels in the posterior eye: a mechanistic model and analytical method. *J Biomech.* 2022; 136: 111052. 35349869 10.1016/j.jbiomech.2022.111052

[123] Avery RL, Castellarin AA, Steinle NC, et al. Systemic pharmacokinetics and pharmacodynamics of intravitreal aflibercept, bevacizumab, and ranibizumab. *Retina.* 2017; 37(10): 1847. 28106709 10.1097/IAE.0000000000001493 PMC5642319

[124] Meyer CH, Krohne TU, Holz FG. Intraocular pharmacokinetics after a single intravitreal injection of 1.5 mg versus 3.0 mg of bevacizumab in humans. *Retina.* 2011; 31(9): 1877–1884. 21738089 10.1097/IAE.0b013e318217373c

[125] Krohne TU, Muether PS, Stratmann NK, et al. Influence of ocular volume and lens status on pharmacokinetics and duration of action of intravitreal vascular endothelial growth factor inhibitors. *Retina.* 2015; 35(1): 69–74. 25077535 10.1097/IAE.0000000000000265

