# Supplementary material for: A First-Passage Model of Intravitreal Drug Delivery and Residence Time—Influence of Ocular Geometry, Individual Variability, and Injection Location
Source: Invest Ophthalmol Vis Sci. 2024 Oct 16;65(12):21. doi: 10.1167/iovs.65.12.21 (PMC11488524; doi:10.1167/iovs.65.12.21)
Supplement: Supplement 1 [file iovs-65-12-21_s001.pdf]

Supplementary Information 1 for "A first passage model of intravitreal drug delivery and residence time - influence of ocular geometry, individual variability, and injection location"

Patricia Lamirande[1], Eamonn A Gaffney[1], Michael Gertz[2], Philip K Maini[1], Jessica R Crawshaw[1], and Antonello Caruso[2]

[1] *Wolfson Centre for Mathematical Biology, Mathematical Institute, Andrew Wiles Building, University of Oxford, Oxford, United Kingdom*

[2] *Pharmaceutical Sciences, Roche Innovation Center Basel, Roche Pharma Research and Early Development, Basel, Switzerland*

S1.1 Derivation of the relation between the mean first passage time and the ocular half-life

The half-life, $t_{1/2}$, is the time required for a quantity that is exponentially decaying to fall to one half of its initial value. In the context of this paper, the ocular $t_{1/2}$ characterises the drug's rate of elimination. In experimental studies, $t_{1/2}$ is usually calculated using the coefficients of the exponential curve fitted to the collected concentration data, using concentration in the aqueous as a proxy for concentration in the vitreous[7]. Here, we derive an equation to link the mean first passage time (MFPT) with $t_{1/2}$.

Let $c(t)$ be the total quantity of drug inside the vitreous at time t, for a specified injection site $\boldsymbol{x_0}$, and c_0, the initial concentration of drug averaged throughout the vitreous. The proportion of drug remaining in the eye at time t is $\frac{c(t)}{c_0}$. Let T, a random variable, be the first passage time for an injection at location $\boldsymbol{x_0}$. Treating all drug molecules as equivalent (so considering the proportion of drug exiting instead of the probability of one particle exiting), we have

$$\text{Prob}(T > t) = \text{Proportion of drug remaining at time } t = \frac{c(t)}{c_0}.$$

Thus

$$\text{Prob}(T < t) = 1 - \frac{c(t)}{c_0},$$

and hence

$$
\begin{aligned}
\text{Prob}(T \in [t, t+\delta t]) &= \text{Prob}((T < t+\delta t) \cap (T \nless t)) \\
&= \text{Prob}(T < t+\delta t) - \text{Prob}(T < t) \\
&= -\frac{1}{c_0}\left(c(t+\delta t) - c(t)\right) \\
&= -\frac{1}{c_0}\frac{dc(t)}{dt}\delta t + \mathcal{O}(\delta t^2),
\end{aligned}
$$

where the last line was obtained using a Taylor approximation around t and δt is a small time increment. Therefore, by taking the limit $\delta t \to 0$, the probability density function for the first passage time T is

$$f_T(t) = -\frac{1}{c_0}\frac{dc(t)}{dt},$$

for the previously specified initial injection location $\boldsymbol{x_0}$. By definition of the mean first passage time, τ is the expected value of the first passage time, so that

$$\tau = \int_0^\infty t\left(-\frac{1}{c_0}\frac{dc}{dt}\right)dt = \frac{1}{c_0}\int_0^\infty c\,dt, \tag{S1.1.1}$$

assuming $c \to 0$ faster than $1/t$ as $t \to \infty$, which is justified as we are expecting a behaviour similar to an exponential decay for $c(t)$.

For $c(t)$ decreasing exponentially, with initial concentration c_0, the drug concentration can be expressed as

$$c(t) = c_0\,\mathrm{e}^{-\lambda t}, \tag{S1.1.2}$$

where λ is the decay rate constant. The corresponding half-life is

$$t_{1/2} = \frac{\ln 2}{\lambda}. \tag{S1.1.3}$$

Using equations (S1.1.2) and (S1.1.3) in equation (S1.1.1), we obtain

$$\tau = \frac{1}{c_0}\int_0^\infty c_0\,\mathrm{e}^{-\lambda t}dt = \frac{1}{\lambda} = \frac{t_{1/2}}{\ln 2}.$$

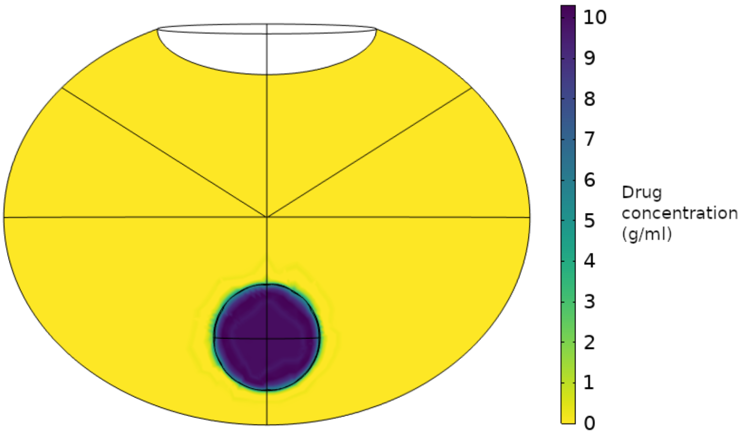

Figure S1.1.1: Initial condition for the diffusion simulation for an injection at the back of the vitreous in the human eye. The parameters used to produce this plot are in Table 1 and Table 3, with the geometry of the human eye illustrated in Figure 2.

Measure	Fab	IgG
MFPT (days)	9.22	15.31
$t_{1/2}$ estimated using the MFPT (days)	6.39	10.61
$t_{1/2}$ estimated from the diffusion simulation and fitted exponential (days)	5.73	9.61
Relative error (%)	11.5	10.4

Table S1.1.1: Relevant quantities for the validation of equation (S1.1.4).

Hence, for a concentration decreasing exponentially at all time, the relation between the MFPT and the ocular half-life is

$$t_{1/2}(\boldsymbol{x_0}) = (\ln 2)\tau(\boldsymbol{x_0}), \tag{S1.1.4}$$

where $\boldsymbol{x_0}$ is the injection location.

To obtain equation (S1.1.4), we made the assumption that $c(t)$ was decreasing exponentially at all time. To support the justification of this assumption, we have solved the diffusion equation for an injection of 0.5 mg of drug in 50 µl liquid[29;28], centered on the optical axis at the back of the vitreous, in the human eye model, for a Fab and an IgG molecule format (see Figure S1.1.1). The details of these simulations (and the material required to reproduce the figures) are in the Github: `https://github.com/patricia-lamy/MFPT-ocular-drug-delivery`. The solutions are illustrated in Figure S1.1.2, where the quantity of injected drug in the vitreous varies with time due to the drug clearance. We fitted an exponential decay function and obtained the decay rate to directly measure the ocular half-life associated with this setting. The logarithmic scale results in Figure S1.1.2 demonstrate how close the exponential fits are to the numerical solutions. The results are summarised in Table S1.1.1.

In Table S1.1.1, the $t_{1/2}$ estimated with the MFPT (using equation (S1.1.4)) was obtained assuming that the

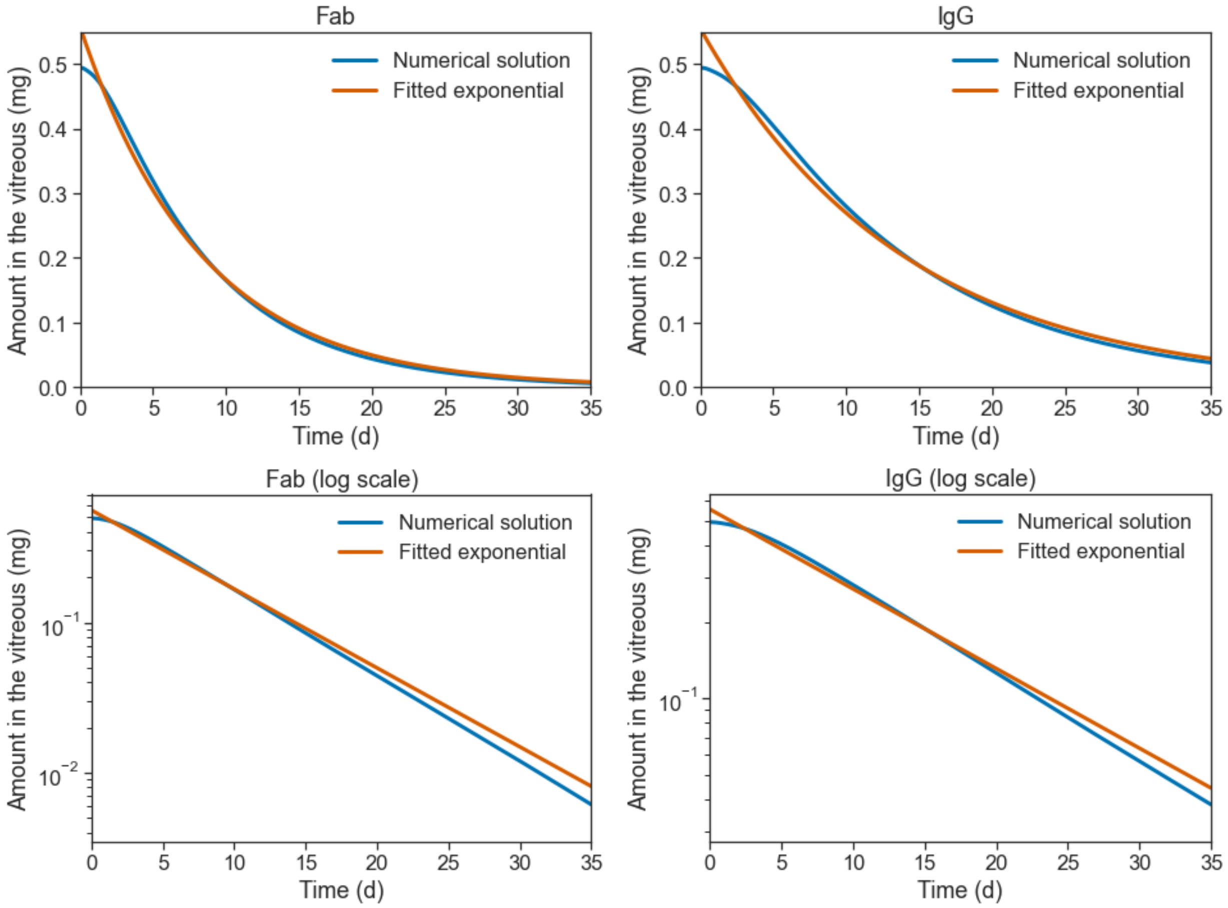

Figure S1.1.2: Numerical solutions of the diffusion simulations for an injection at the back of the eye in the human eye, for a Fab (left) and an IgG (right) molecule, with the fitted exponential decay function used to calculate directly the ocular half-life. The second row shows the results on a logarithmic scale, to better compare the exponential fits.

quantity of drug leaving the vitreous followed an exponential decrease, whereas the second $t_{1/2}$ was obtained by fitting an exponential function to the decrease of drug quantity over time. In experimental settings, where the quantity or concentration of drug is measured over time, the half-life is obtained by the second method, i.e. by fitting an exponential function and extracting its decay rate. Hence, we considered the $t_{1/2}$ derived by the diffusion simulation to be more representative of the experimentally measured $t_{1/2}$. For an injection site at the back of the eye (which provided the largest discrepancy), we obtained differences of 10.4% and 11.5% between the two measures, for an IgG and a Fab molecule respectively. Considering the high uncertainty on the permeability parameters, obtained from rabbit data, we did not expect our model to have the ability of estimating the ocular half-lives with a great precision and consider a 10% relative error introduced by our modelling framework to be acceptable.

S1.2 Details on geometry construction

Details on geometry construction for each species

Below is a detailed description of the construction of the canonical eye model for each species, as illustrated in Figure 2 and Figure 3 of the main text, with parameters specified in Table 3.

Human

Given experimental data for eye geometries as a function of age, we chose to consider measures for the range of 50-95 years old, to reflect the age range of the majority of people affected by wet AMD. Using measures from the literature, canonical parameters are given as follows:

- The vitreous chamber diameter was set to 2.255 cm[1], taking the average height and width measures from Table 1 in Atchison et al.[1] for emmetropic eyes, which yields a semi-axis of $a = 1.1275$ cm for the ellipsoid representing the vitreous chamber.
- The lens thickness was set to 0.3909 cm, using a linear fit for 50 year-olds from MRI measures[20].
- For 50 year-olds, with the linear regression from Rosen et al.[34], the lens diameter was estimated to be 0.939 cm.
- Based on in situ MRI, we set $l_p = 50\%$, i.e. we supposed that half of the lens is situated inside the vitreous chamber cavity[1].

- The optical axial length denotes the length between the retina and the cornea on the optical axis and was set to 2.30 cm, the average measure for emmetropic eyes in Atchison et al.[1]. The anterior chamber depth, that is the length between the cornea and the lens, was set to 0.3276 cm[20], using the citation's linear fit for 50 year-olds from MRI measures. We defined the semi-axis b as half the length on the optical axis between the centre of the lens and retina. Subtracting the anterior chamber depth and the anterior half of the lens thickness from the axial length, we obtained

$$b = \frac{2.30 - (0.3276 + 0.3909/2)}{2} \text{ cm} = 0.889 \text{ cm}.$$

- For the height of the vitreous-aqueous interface, we used the estimated ratio of vitreous-aqueous surface area to the total surface area of 15% to define $h_{va} = 0.251$ cm[16].

We validated these ocular dimensions by comparing the vitreous volume and the retinal surface area with measures from the literature. The canonical model's geometry had a vitreous volume of 4.595 ml, which was in the range of vitreous volumes measured for 50 to 95-year-olds[3]. The constructed geometry had a retinal surface area of 10.963 cm^2, which was within the range of retinal surface areas measured in the literature. Finally, we validated the geometry by comparing it with an in situ MRI of a human eye, as illustrated in Figure 3.

Cynomolgus monkey

- The lens diameter was set to 0.75 cm, taking the mean of the second group of cynomolgus monkeys considered by Manns et al.[24], which included lenses from 'older donors'.
- The lens thickness was set to 0.351 cm, taking the mean value of Choi et al.[8].
- Based on the longitudinal section of a cynomolgus monkey eye, we set the proportion of the lens inside the vitreous chamber cavity l_p to be 50%[40].
- The anterior chamber depth was set to 0.309 cm[8], and the optical axial length was set to 1.841 cm[8]. The semi-axis b for the vitreous chamber ellipsoid was obtained by subtracting the anterior chamber depth and half of the lens thickness from the optical axial length, i.e.

$$b = \frac{1.841 - (0.309 + 0.351/2)}{2} \text{ cm} = 0.678 \text{ cm}.$$

- The height of the vitreous-aqueous interface was set to 0.163 cm, so that the ratio of the surface of

the vitreous-aqueous humour interface to the total surface of the vitreous ellipse was approximately 13%[16].

- No experimental measure of the vitreous chamber diameter of the cynomolgus monkey was found in the literature in order to parameterise a. We therefore used the measure of the vitreous volume from the literature to fix a. In order to have a vitreous volume value of $V_{\text{vit}} = 2.2$ cm^3, we set $a = 0.895$ cm[2]. To fit the range of vitreous volumes of 2.0 to 2.3 ml from Atsumi et al.[2], we set the range of $a \in [0.855, 0.915]$ cm.

In contrast with the other species, we could not use the vitreous volume to validate our ocular dimensions, as we used the literature vitreous volume to define the semi-axis of the vitreous chamber width a. Therefore, we validated the constructed geometry by comparing the model's retinal surface area with measures from the literature. The geometry had a retinal surface area of 6.9105 cm^2, which was within the range of retinal surface areas reported in the literature for the rhesus monkey[44] (no measure could be found for the cynomolgus monkey), which ranged between 5.8 and 9.2 cm^2, with a mean of 7.30 cm^2. The rhesus monkey eyes are similar to the cynomolgus monkey eyes, with a slightly larger axial length (between 1.9 cm and 2.0 cm)[11]. Finally, we validated the geometry by comparing it with a sectional image of a cynomolgus monkey eye, as illustrated in Figure 3 of the main text.

Rabbit

- In contrast to the human and cynomolgus monkey eyes, the rabbit lens has more than half of its volume inside the vitreous chamber (see Figure 3, or see MRI of rabbit eyes in the literature[37;42]). Guided by in situ MRI (see Figure 3), we applied a translation of the centre of the lens of $l_T/7$ towards the centre of the vitreous ellipse, to obtain a geometry that visually matched, with approximately 2/3 of the lens inside the vitreous chamber.

- The lens thickness was set to 0.66 cm, the mean value of Atsumi et al.[2]. Its range was determined by the range of measures reported in Atsumi et al.[2] and Liu and Farid[21].

- The lens diameter was set to 0.995 cm, the mean value of in situ measurements in Werner et al.[43], and its range to the standard deviation reported in the manuscript.

- The anterior chamber depth was set to 0.234 cm[21]. The optical axial length was set to 1.631 cm[21]. We set the semi-axis b as half the length on the optical axis between the retina and the portion of the lens inside the vitreous chamber. The semi-axis b was obtained by using the lens thickness (with the assumption that 1/3 of the lens thickness in outside of the vitreous), the anterior chamber depth, and

the optical axial length:

$$b = \frac{1.631 - (0.234 + 0.66/3)}{2}\ \text{cm} = 0.588\ \text{cm}.$$

We defined the range for the semi-axis b using the minimum and maximum values for the axial length, the anterior chamber depth, and the lens thickness. Liu et al.[21] measured a range of [0.230, 0.253] cm for the anterior chamber depth of rabbits, and a range of [1.618, 1.672] cm for the axial length. From these results, we obtained the lower and greater bounds for the range of the semi-axis b:

$$b_{\min} = \frac{1.618 - (0.253 + 0.697/3)}{2}\ \text{cm} = 0.566\ \text{cm},$$
$$b_{\min} = \frac{1.672 - (0.230 + 0.66/3)}{2}\ \text{cm} = 0.611\ \text{cm}.$$

- The vitreous diameter was set to 1.8 cm, using the mean value measured in Sawada et al.[37], and its range determined from using the standard deviation from the mean reported in this citation, rendering a semi-axis estimate of $a = 0.90$ cm.

- The height of the vitreous-aqueous interface was set to 0.238 cm, so that the ratio of the surface of the vitreous-aqueous humour interface, with the total surface of the ellipse approximately 23%[16].

The model's geometry for the rabbit had a vitreous volume of 1.7078 ml, which fell within the range of vitreous volumes measured in the literature (1.15-1.8 ml). The constructed geometry had a retinal surface area of 5.4367 cm^2, which was within the range of 4 to 6 ml measured experimentally[33]. Finally, we validated the geometry by comparing it with an in situ MRI of a rabbit eye, as illustrated in Figure 3.

Rat

When possible, we considered measures for adult rats (120 days-old or older) to inform the model's construction.

- We assumed that the lens was almost entirely immersed in the vitreous chamber cavity, with only a small cap emerging in the anterior chamber. Guided by in situ MRI[9], we applied a translation of length $l_T/4$ of the lens centre towards the centre of the vitreous ellipse to achieve a similar geometry, so that a small cap of the lens emerged from the vitreous chamber. For simplicity, in order to define the parameter values to construct the geometry, we considered the lens thickness to be entirely inside the vitreous chamber.

- The lens thickness was set to 0.387 cm, the mean from Massof and Chang[26], and its range was set using the mean measurements from Hughes[14] and Lozano and Twa[22].

- The lens diameter was set to 0.432 cm, the mean from Massof and Chang[26], and its range was set using the mean measurements from Hughes[14] and Pe'er et al.[32].

- The optical axial length was set to 0.572 cm, taking the axial length from Hughes[14] without the corneal, retina, choroid and scleral thickness measures. The anterior chamber depth was set to 0.062 cm[14]. As we assumed that the lens was entirely inside the vitreous chamber, we did not need to subtract a portion of the lens thickness from the axial length (as we did for the previous species). This yielded a semi-axis of length

$$b = \frac{0.572 - 0.062}{2} \text{ cm} = 0.255 \text{ cm}.$$

  We defined the range of values for b using the standard deviation identified for the axial length in Hughes[14].

- The vitreous diameter was set to 0.579 cm, taking the measure of the eye width from Hughes[14], and subtracting from it the retinal, choroid and scleral thickness on both sides of the diameter. This yielded a semi-axis of length $a = 0.2895$ cm in the model. We obtained the range of values for a by taking the standard deviation of the vitreous diameter reported in Hughes[14].

- The height of the vitreous-aqueous interface was initially determined by fitting our model to the in vivo MRI of a rat[9] (see Figure 3), using the visible ciliary body as the end of the retina, which suggested $h_{va} = 0.08$ cm. This yielded a surface ratio of 27.42% for the vitreous-aqueous interface over the total area of the vitreous ellipsoid, and a retinal surface area of 0.64813 cm^2. As the retinal surface area we obtained was less than the estimated areas from the literature[27;4] (ranging from 0.65 cm^2 to 0.8 cm^2), we set $h_{va} = 0.07$ cm, to have a retinal surface area of 0.667 cm^2. Doing this, we had a retinal surface area that fell inside the range of values identified from the literature, and a model that visually matched the in situ MRI.

The model's geometry had a vitreous volume of 51.827 µl, which was close to the vitreous volume of 52.4 µl (±1.9 µl) estimated for 120 day-old rats[39]. The retinal surface area also lay within the literature range, as it was used to define h_{va}. Finally, we validated the geometry by comparing it with an in situ MRI of a rat eye, as illustrated in Figure 3.

Mouse

Given experimental data for murine eyes as a function of age, we chose to consider measures for mice of approximately 3 months old. This was guided by the aim to have a model to compare with experimental results from 8-week-old mice[6], and constrained by the availability of measurements in the literature. All ocular dimensions considered were measured on mice of strain C57/BL6.

- Similar to the rat, the mouse lens is almost entirely situated in the vitreous chamber cavity, with only a small cap emerging in the anterior chamber. Guided by in situ MRI[18;31;38;41], we applied a translation of a distance $l_T/4$ of the lens' centre towards the centre of the vitreous ellipse to achieve a similar geometry, so that a small cap of the lens emerged from the vitreous chamber. For simplicity, in order to define the rest of the parameters to construct the geometry, we supposed that the lens thickness was entirely inside the vitreous chamber.
- The vitreous chamber diameter was set to 0.3236 cm ($a = 0.1618$ cm), taking the mean vitreous chamber diameter for mice aged 89 days[41].
- Inferred from the linear regression and data points for 3-month-old mice from Schmucker and Schaeffel[38], the anterior chamber depth was set to 0.0362 cm and the axial length was set to 0.3073 cm (based on the axial length measure, from which we subtracted the corresponding retinal thickness). Supposing that the entire lens thickness was within the vitreous body, we obtained the semi-axis b by subtracting the anterior chamber depth from the axial length:

$$b = \frac{0.3073 - 0.03623}{2} = 0.1355 \text{ cm.}$$

- We set the lens diameter and thickness by slightly adjusting the values found in the literature to fit the lens volume to $6.50\,\mu$l for 3-month-old mice[31]. As there was a discrepancy between the volume and the measure of the lens' axes in our calculations, we decided to use the volume as reference, as it led to the best visual match with the in situ MRI (Figure 3). It was reported that mice had lens diameters of approximately 0.225 cm for 3-month-old mice, and lens thicknesses of approximately 0.198 cm[31]. We incrementally increased these values until we obtained a lens volume close to the one found in the literature, with the constraint that the lens thickness should be less than the lens diameter, and validating the results with the in situ MRI image (Figure 3). We obtained:

$$l_D = 0.240 \text{ cm}$$

$$l_T = 0.216 \text{ cm.}$$

- A first attempt to define the height of the vitreous-aqueous interface was made by fitting our model to in vivo MRI (Figure 4B from Schmucker and Schaeffel[38]), and resulted in $h_{va} = 0.04$ cm. This corresponded to a surface ratio of 25% for the vitreous-aqueous interface (compared to the total surface of the vitreous chamber ellipsoid), and a retinal surface area of 0.199 cm^2. As the retinal surface area exceeded the range of measurements found in the literature, we incrementally increased h_{va} until $h_{va} = 0.05$ cm, which yielded a retinal surface area of $A_{\text{ret}} = 0.188\ \text{cm}^2$.

The model's geometry had a vitreous volume of 8.42 µl, which was in the range of the vitreous volume measurements from the literature, spanning 4.4 to 12 µl. As mentioned, the retinal surface area measurements from the literature was used to refine the geometry by adjusting h_{va}, so surface area comparisons are not feasible. Finally, we validated the geometry by comparing it with an in situ MRI of a mouse eye, as illustrated in Figure 3.

Details on the construction of the ensemble of human eye geometries

We used the data and the results of experimental studies to build an ensemble of human eye dimensions. In most cases, we used the axial length and the vitreous volume measures to reconstruct the eyes, under the assumption of constant anterior chamber depth and lens thickness, and assuming that the eye is axisymmetric around the optical axis. We considered the assumption of a constant anterior chamber depth to be reasonable, based on a weak correlation between the anterior chamber depth and the axial length[45], and based on the high individual variability of the anterior chamber depth between individuals within the same refractive error group[12]. While a correlation has been identified between the lens thickness and the axial length[30], the reported variability of the lens thickness associated with the axial length is no greater than observed variations of lens thickness found in the population in general (regardless of axial lengths), for example in relation to lens thickness variation with age[34]. Regardless, by varying the axial length, we obtained a range of eye dimensions covering the variability for the lens thickness and anterior chamber depth.

In all cases, we used the same method as described in Section 2 for the human eye to obtain a value of b from the axial length measurement. When no measurement for the vitreous diameter was provided, we used the provided vitreous volume to obtain a, with the assumption that the volume of the vitreous chamber ellipsoid formed by a and b is the combination of the vitreous volume and half of the lens volume. The different sources used different measurement and estimation methods, which are summarised in Table S1.2.1.

We directly used the measurements from Atchison et al.[1]. From their Table 1, we took the average measurement for the height (vitreous diameter measured in the sagittal plane) and the width (vitreous diameter measured in the axial plane) as the vitreous diameter to obtain a, and we took the average length between the axial and sagittal image for the axial length to obtain b. We used the digitised measurements of axial lengths and vitreous volumes from the figures presented in Azhdam et al.[3], de Santana et al.[10], and Zhou et al.[45] to build the rest of the eye geometries. For Zhou et al.[45], we only kept the data for pathological myopia, as there may be a discrepancy between the figure for emmetropic axial length and volume (Figure 2 of Zhou et al.[45]) and their mean and slope specified in the main text (section 3.3 of Zhou et al.[45]). The digitised data and the eye measurements of the ensemble of human eyes are provided in Supplementary 2 and in the Github repository `https://github.com/patricia-lamy/MFPT-ocular-drug-delivery`. After digitising the data and taking the mean measurements available from Atchison et al.[1], we obtained an ensemble of 155 human eye models.

Source	Sample size	Inclusion criteria	Refractive error range	Measurement of AL	Measurement of volume (or other measurements)
Atchison et al. (2004) [1]	88 eyes	Aged between 18 and 36 years, good ocular health.	Emmetropic and myopic.	MRI	Other ocular measurements (height, width of vitreous volumes) were estimated by MRI.
Azhdam et al. (2020) [3]	100 eyes	No ocular pathology and no history of ocular surgery.	Not specified.	Estimated from CT scan.	Volume estimated by CT scan with Mimics image analysis tool to estimate vitreous volume.
Zhou et al. (2020) [45]	290 eyes	Met the diagnostic criteria of pathological myopia, aged between 18 and 60 years, no history of ocular diseases affecting diopter, and no history of ocular surgery.	Pathological myopia.	Optical biometry.	Volume estimated by MRI.
de Santana et al. (2021) [10]	112 eyes	Pseudophakia, aged older 50 years, eyes with axial length between 2.1 to 2.6 cm.	Not specified.	Optical biometry	Vitrectomy and, after the fluid-air exchange, the vitreous chamber was filled with a dye. The infused volume of each eye was recorded.

Table S1.2.1: Description of sources, methods, and measurements used to build the ensemble of human eyes.

S1.3 Results of the global sensitivity analysis on the MFPT

As described in the Methods section of the manuscript, a global sensitivity analysis was carried out in order to assess the effect of the parameters of the model on the MFPT for an injection at location P_m. In the human, cynomolgus monkey and rabbit eye, P_m corresponds to the midpoint of the vitreous chamber depth along the optical axis (see Figure 2). The sensitivity analysis was performed using the eFAST sensitivity method[36;25], a variance-based method yielding the same sensitivity indices as the Sobol' indices, but in a more computationally efficient manner[35]. This was implemented with the python SAlib library[17;13].

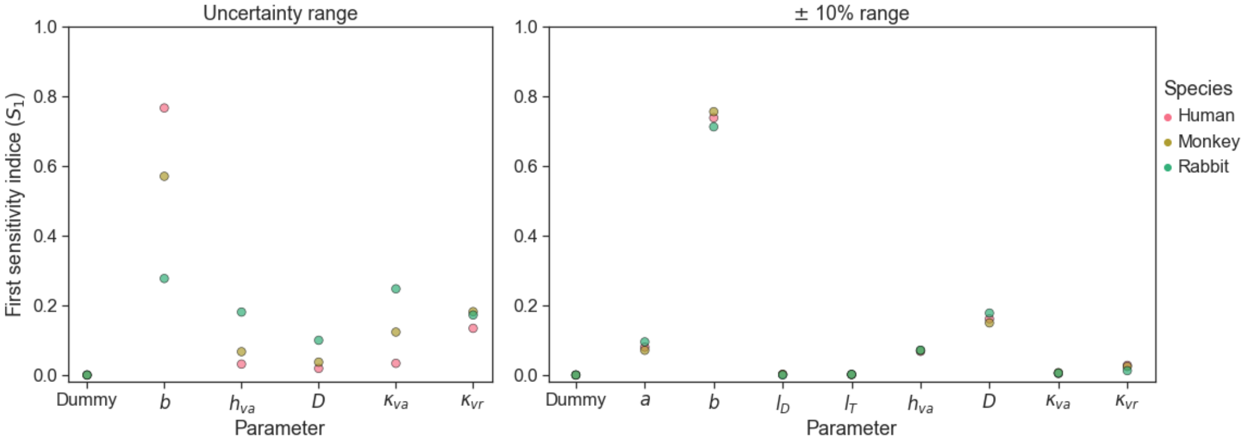

Figure S1.3.1: Results for the global sensitivity analysis of the MFPT for a Fab molecule for an injection location at P_m, for the human, cynomolgus monkey, and rabbit eye models. On the left, the parameters were varied within their identified uncertainty range (see Table 3 for the geometrical parameters and Table S1.3.1 for the drug-dependent parameters), and on the right, the parameters were varied within a ±10% range around their model value (see Table 1 and 2). The semi-axes a and b are, respectively, the semi-major and semi-minor axis of the vitreous chamber ellipse, l_D and l_T are the lens diameter and thickness, and h_{va} is the height of the vitreous-aqueous humour interface, as defined in Figure 1 of the main text. The drug-dependent parameters are the diffusion coefficient D, and the permeability parameters for the vitreous-aqueous humour interface and vitreous-retina interface are κ_{va} and κ_{va}, respectively.

In order to choose the right number of samples to generate, N_s, and other algorithm parameters, a convergence study was performed for each set of parameters' range, where the sensitivity indices for an increasing large N_s were compared. Two sets of values for the nine model parameters were investigated, to assess the model's sensitivity to the uncertainty of the parameters (Figure S1.3.1 left side), and to assess the relative influence of the parameters on the model's results (Figure S1.3.1 right side). In the first case, for the parameters' uncertainty range, convergence of the results could not be obtained for all nine parameters varying. Therefore, the sensitivity analysis was first performed on the geometrical parameters, where the non-influential parameters were set to fixed values before repeating the sensitivity analysis with the remaining parameters. Based on our convergence analysis, the number of samples needed was set to $N_s = 337$,

Drug-dependent parameter	Uncertainty range	Source
Diffusion coefficient (D)	$(1.01, 1.13) \times 10^{-6}$ cm^2/s	Caruso et al.[7]
Permeability of vitreous-aqueous humour interface (κ_{va})	$(1.24, 3.92) \times 10^{-5}$ cm/s	Hutton-Smith[15]
Permeability of vitreous-retina interface (κ_{vr})	$(1.25, 2.44) \times 10^{-7}$ cm/s	Hutton-Smith[15]

Table S1.3.1: Uncertainty ranges used in the global sensitivity analysis for the drug-dependent parameters of a Fab molecular format.

the number of harmonics to sum in the Fourier series decomposition was set to $M = 4$, and the maximum frequency was set to $\omega_{\text{max}} = 42$. The implementation of the sensitivity analysis sampling was validated by confirming that a dummy variable has sensitivity indices of around zero, demonstrating minimum sampling artefact[25].

Figure S1.3.1 shows the first order sensitivity indices, for a Fab molecule format injected at the injection point P_m, for two sets of parameter values. The total sensitivity indices are not illustrated, as they essentially do not differ from the first sensitivity indices.

On the left-hand side of Figure S1.3.1, the parameter values were varied within their uncertainty range for each species: b was varied within the range of ocular values in the literature identified in Table 1, h_{va} within $\pm 10\%$ of its base value (Table 1), and the drug-dependent parameters within the range identified in Table S1.3.1. The geometrical parameters a, l_D and l_T (see Figure 1 for their definition) are not illustrated in the left-hand side of Figure S1.3.1, but were revealed to be of little influence (sensitivity indices <0.05, result not shown). On the right-hand side of Figure S1.3.1, parameters were varied within $\pm 10\%$ of their model value (see Table 1 and Table 2).

The global sensitivity analysis identified that, within the uncertainty range of each parameter and for an injection at P_m, the length of the semi-axis b, as depicted in Figure 1 of the main text, was the most sensitive for the MFPT. The global sensitivity analysis also revealed that the model is not inherently sensitive to the permeability parameters, as their sensitivity indices were low when they varied within $\pm 10\%$ of their values.

S1.4 Derivation of the mean first passage time for a bolus

Let Y be a random variable describing the initial position of a particle in a sphere V_b of radius r_b, with Y following a uniform distribution. Following the law of total probability,

$$\text{Prob}[T(Y) \leq t] = \frac{1}{\text{Vol}(V_b)} \int_{V_b} \text{Prob}\left[T(\boldsymbol{y}) \leq t\right] dV.$$

Introducing the survival probability $\mathbb{P}(\boldsymbol{y}, t)$ as the probability that the particle starting at $\boldsymbol{y}$ has not yet exited the domain by time t, it follows that[5]

$$\text{Prob}[T(\boldsymbol{y}) \leq t] = 1 - \mathbb{P}(\boldsymbol{y}, t),$$

and the density function of the above probability distribution is given by

$$f(\boldsymbol{y}, t) = \frac{\partial}{\partial t}\text{Prob}[T(\boldsymbol{y}) \leq t] = \frac{-1}{\text{Vol}(V_b)} \int_{V_b} \frac{\partial \mathbb{P}(\boldsymbol{y}, t)}{\partial t}\, dV.$$

Finally, using the definition of the mean first passage time (MFPT)

$$\tau_b(Y) = \mathbb{E}[T(Y)]\,, \text{ and } \tau(\boldsymbol{y}) = \int_0^t t\, f(\boldsymbol{y}, t) dt.$$

It follows that

$$\tau_b(Y) = \int_0^t t \frac{\partial}{\partial t}\text{Prob}[T(\boldsymbol{y}) \leq t] = \frac{1}{\text{Vol}(V_b)} \int_{V_b} \tau(\boldsymbol{y}) dV, \qquad \text{(S1.4.1)}$$

where $\tau_b(Y)$ is defined as the MFPT for the bolus. With the result of equation (S1.4.1), the following proposition estimates the impact of an injection bolus on the calculations of the MFPT from a specific injection point.

Proposition 1. *Under the assumption that $\tau(\boldsymbol{y})$ possesses a convergent Taylor series within the region of the injection bolus, the MFPT for a particle starting in the sphere V_b of radius r_b centered on $\boldsymbol{y_b}$ can be expressed as*

$$\frac{1}{Vol(V_b)} \int_{V_b} \tau(\boldsymbol{y}) dV = \tau(\boldsymbol{y_b}) - \frac{{r_b}^2}{10\, D},$$

where D is the diffusion coefficient associated with the MFPT.

Proof. Under the assumption that $\tau(\boldsymbol{y})$ possesses a convergent Taylor series within the region of the injection

bolus, the MFPT around the bolus centre $\boldsymbol{y_b}$ can be expressed as

$$\tau(\boldsymbol{y}) = \sum_{|\alpha|=0}^{\infty} \frac{1}{\alpha_1!\alpha_2!\alpha_3!}\left(\frac{\partial^{|\alpha|}\tau}{\partial {y_1}^{\alpha_1}\partial {y_2}^{\alpha_2}\partial {y_3}^{\alpha_3}}(\boldsymbol{y_b})\right)(\boldsymbol{y}-\boldsymbol{y_b})_1^{\alpha_1}(\boldsymbol{y}-\boldsymbol{y_b})_2^{\alpha_2}(\boldsymbol{y}-\boldsymbol{y_b})_3^{\alpha_3},$$

where we defined $|\alpha| = \alpha_1 + \alpha_2 + \alpha_3$. We are interested in estimating equation (S1.4.1), and thus in simplifying $\int_{V_b} \tau(\boldsymbol{y})dV$. By symmetry of the sphere V_b around $\boldsymbol{y_b}$, we have by parity that, for $|\alpha|$ odd,

$$\int_{V_b} (\boldsymbol{y}-\boldsymbol{y_b})_1^{\alpha_1}(\boldsymbol{y}-\boldsymbol{y_b})_2^{\alpha_2}(\boldsymbol{y}-\boldsymbol{y_b})_3^{\alpha_3}\, dV = 0\,.$$

Let $|\alpha|$ be even. We define a new notation: let $i_1, i_2, \ldots i_{|\alpha|}$ be the list of indices of the linear combination, where they can correspond to each of the Cartesian coordinates i, j, k, and where they can be repeated. Then, as every isotropic tensor of even rank can be expressed as a linear combination of products of Kronecker deltas δ_{ij}, δ_{km}, etc.[19], it follows that

$$\int_{V_b} (\boldsymbol{y}-\boldsymbol{y_b})_{i_1} \ldots (\boldsymbol{y}-\boldsymbol{y_b})_{i_{|\alpha|}}\, dV = A\,\delta_{i_1 i_2}\delta_{i_3 i_4}\delta_{i_5 i_6}\ldots + B\,\delta_{i_1 i_3}\delta_{i_2 i_4}\delta_{i_5 i_6}\ldots + \ldots\,,$$

where $A, B, \ldots$ are coefficients, δ_{ij} are the Kronecker deltas, and the summation is over all possible permutations of the indices.

Hence, for $|\alpha| \geq 4$ even,

$$\left(\frac{\partial^{|\alpha|}\tau}{\partial y_{i_1}\ldots y_{i_{|\alpha|}}}(\boldsymbol{y_b})\right)\int_{V_b} (\boldsymbol{y}-\boldsymbol{y_b})_{i_1}\ldots(\boldsymbol{y}-\boldsymbol{y_b})_{i_{|\alpha|}} dV = \frac{\partial^{|\alpha|-2}}{\partial y_{i_3}\ldots\partial y_{i_n}}\frac{\partial^2\tau}{\partial y_{i_1}^2}(\boldsymbol{y_b})(A\,\delta_{i_3 i_4}\ldots+\ldots)\,,$$

and thus

$$\begin{aligned}\sum_{|\alpha|\geq 4}\left(\frac{\partial^{|\alpha|}\tau}{\partial y_{i_1}\ldots y_{i_{|\alpha|}}}(\boldsymbol{y_b})\right)\int_{V_b} (\boldsymbol{y}-\boldsymbol{y_b})_{i_1}\ldots(\boldsymbol{y}-\boldsymbol{y_b})_{i_{|\alpha|}} dV &= \frac{\partial^{|\alpha|-2}}{\partial y_{i_3}\ldots\partial y_{i_n}}\nabla^2\tau(\boldsymbol{y_b})(A\,\delta_{i_3 i_4}\ldots+\ldots)\,,\\ &= 0\,,\end{aligned}$$

as, by definition of the MFPT, $\nabla^2\tau(y_b) = -1/D$, a constant, which is annihilated by the further derivatives.

It follows that

$$\frac{1}{\mathrm{Vol}(V_b)}\int_{V_b}\tau(\boldsymbol{y})dV = \tau(\boldsymbol{y_b}) + \frac{1}{2}\frac{\partial^2\tau}{\partial y_{i_1}\partial y_{i_2}}(\boldsymbol{y_b})\,\frac{1}{\mathrm{Vol}(V_b)}\int_{V_b}(\boldsymbol{y}-\boldsymbol{y_b})_{i_1}(\boldsymbol{y}-\boldsymbol{y_b})_{i_2}dV,$$

because all other terms of the sum are zero, as shown above. Using again the fact that isotropic tensors of

even rank can be expressed as a linear combination of Kronecker deltas[19], we have that

$$\int_{V_b} (\boldsymbol{y} - \boldsymbol{y_b})_{i_1} (\boldsymbol{y} - \boldsymbol{y_b})_{i_2} dV = \lambda \delta_{i_1 i_2},$$

with λ a constant coefficient. Without loss of generality, where we use $z := (\boldsymbol{y} - \boldsymbol{y_b})_3$, and r, θ, and ϕ are the corresponding spherical coordinates,

$$\lambda = \int_{V_b} z^2 dV = \int_0^{2\pi} \int_0^{\pi} \int_0^{r_b} (r^2 \cos^2\theta)\, r^2 \sin\theta \, d\phi \, d\theta \, dr = 2\pi \frac{{r_b}^5}{5} \left(-\frac{1}{3} \cos^3\theta \right) \Big|_0^{\pi} = \frac{4\pi}{15} {r_b}^5.$$

It follows that

$$\frac{1}{\mathrm{Vol}(V_b)} \int_{V_b} \tau(\boldsymbol{y}) dV = \tau(\boldsymbol{y_b}) + \frac{1}{2} \frac{3}{4\pi {r_b}^3} \frac{4\pi {r_b}^5}{15} \nabla^2 \tau(\boldsymbol{y_b}) = \tau(\boldsymbol{y_b}) + \frac{{r_b}^2}{10} \left(-\frac{1}{D} \right) = \tau(\boldsymbol{y_b}) - \frac{{r_b}^2}{10D},$$

as required.

□

Hence, the MFPT for the bolus Y, defined as a sphere of radius r_b centered at $\boldsymbol{y_b}$, is

$$\tau_b(Y) = \tau(\boldsymbol{y_b}) - \frac{{r_b}^2}{10\,D}.$$

For $r_b = 0.2285$ cm, which corresponds to the standard dose volume of 0.5 ml for ranibizumab intravitreal injections[23],

$$\frac{{r_b}^2}{10\,D} = 0.056 \text{ days},$$

for $D = 1.07 \times 10^{-6}$ cm^2/s. For $r_b = 0.3752$ cm, the radius of the injection location region for the human eye (see Figure 2 of the manuscript), the MFPT of the bolus is

$$\frac{{r_b}^2}{10\,D} = 0.15 \text{ days}.$$

Hence, for the scale of injection regions we are interested in, the MFPT at the centre of the bolus is a good estimate of the MFPT for the surrounding region, considering the MFPT ranges between 6 and 9 days in the posterior section of the vitreous chamber. This result is limited to injection locations sufficiently away from the boundaries, due to the assumption that the injected solution is in the shape of a sphere. The result has been validated using COMSOL, where the average MFPT in the injection volume could be directly calculated using numerical methods.

S1.5 Additional figures

Results of the ensemble of human eye models, excluding pathologically myopic eyes

Figure S1.5.1 shows the MFPT in the ensemble of human eyes without the pathological myopia dataset, plotted against the axial length (AL) and the vitreous volume.

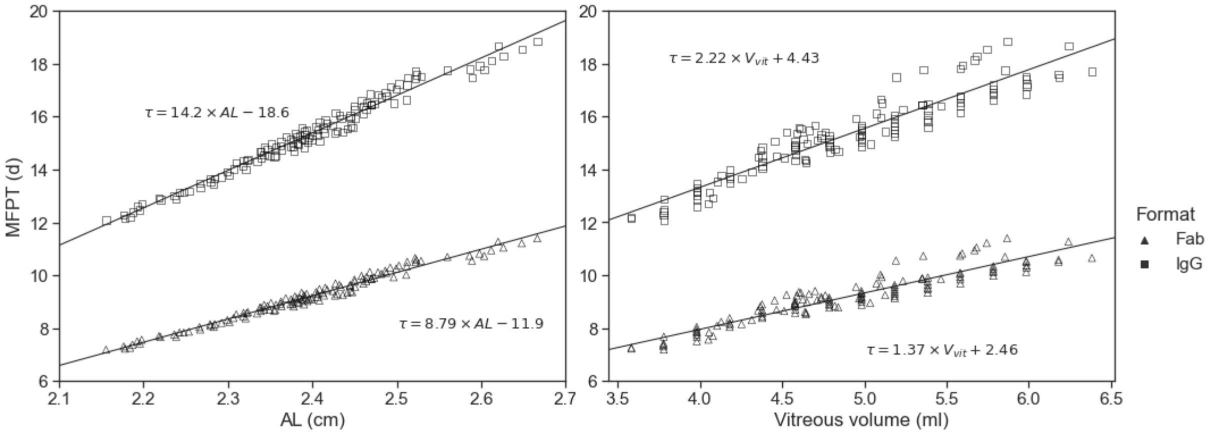

Figure S1.5.1: Numerical solution and linear regressions of the MFPT for an injection at P_m, for different molecular formats and with parameters defined in Table 1, for the ensemble of human eye models without pathology, plotted against the axial length (AL) and the vitreous volume.

Conditional MFPT

To obtain numerical solutions for the conditional MFPT, equations (2.3) and (2.4) were solved with parameter values given in Table 1 for a Fab molecule, using the eye geometry for the cynomolgus monkey, rabbit, rat and mouse (Figure 2). Figure S1.5.2 shows the results for the conditional MFPT for particles exiting through the vitreous-retina and vitreous-aqueous humour interfaces.

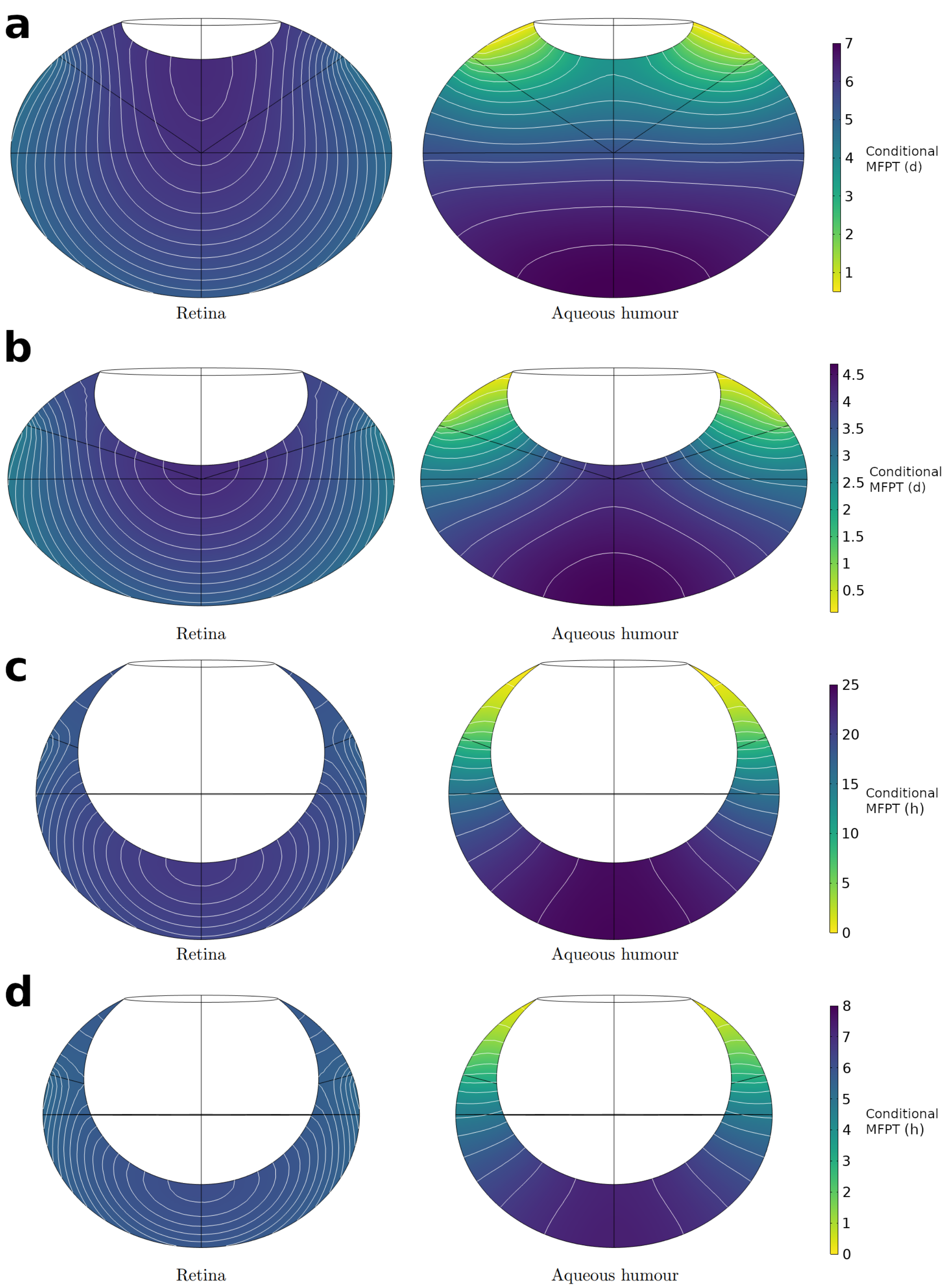

Figure S1.5.2: Numerical solution and contour lines for the MFPT, conditional on exiting through the vitreous-retina and vitreous-aqueous humour interfaces for a Fab molecule as a function of injection site, for a) cynomolgus monkey, b) rabbit, c) rat and d) mouse eye models. The parameters for these plots are in Table 1 and Table 3, and the geometries used are illustrated in Figure 2.

References

[1] D. A. Atchison, C. E. Jones, K. L. Schmid, N. Pritchard, J. M. Pope, W. E. Strugnell, and R. A. Riley. Eye shape in emmetropia and myopia. *Investigative Ophthalmology & Visual Science*, 45(10):3380–3386, 2004.

[2] I. Atsumi, M. Kurata, and H. Sakaki. Comparative study on ocular anatomical features among rabbits, beagle dogs and cynomolgus monkeys. *Anim. Eye Res.*, 32:35–41, 2013.

[3] A. M. Azhdam, R. A. Goldberg, and S. Ugradar. In vivo measurement of the human vitreous chamber volume using computed tomography imaging of 100 eyes. *Translational Vision Science & Technology*, 9(1):2–2, 2020.

[4] T. Baden, T. Euler, and P. Berens. Understanding the retinal basis of vision across species. *Nature Reviews Neuroscience*, 21(1):5–20, 2020.

[5] P. C. Bressloff and J. M. Newby. Stochastic models of intracellular transport. *Reviews of Modern Physics*, 85(1):135–191, 2013.

[6] D. Bussing, Y. Li, L. Guo, A. Verma, J. M. Sullivan, and D. K. Shah. Pharmacokinetics of monoclonal antibody and antibody fragments in the mouse eye following intravitreal administration. *Journal of Pharmaceutical Sciences*, 2023.

[7] A. Caruso, M. Füth, R. Alvarez-Sánchez, S. Belli, C. Diack, K. F. Maass, D. Schwab, H. Kettenberger, and N. A. Mazer. Ocular half-life of intravitreal biologics in humans and other species: meta-analysis and model-based prediction. *Molecular Pharmaceutics*, 17(2):695–709, 2020.

[8] K.-E. Choi, V. T. Q. Anh, C. Yun, Y.-J. Kim, H. Jung, H. Eom, D. Shin, and S.-W. Kim. Normative data of ocular biometry, optical coherence tomography, and electrophysiology conducted for cynomolgus macaque monkeys. *Translational Vision Science & Technology*, 10(13):14–14, 2021.

[9] T. Y. Chui, D. Bissig, B. A. Berkowitz, and J. D. Akula. Refractive development in the "ROP rat". *Journal of Ophthalmology*, 2012, 2012.

[10] J. M. de Santana, G. G. Cordeiro, D. T. C. Soares, M. R. Costa, A. Paashaus da Costa Pinto, and R. P. C. Lira. Use of axial length to estimate the vitreous chamber volume in pseudophakic. *Graefe's Archive for Clinical and Experimental Ophthalmology*, 259:1471–1475, 2021.

[11] A. Fernandes, D. V. Bradley, M. Tigges, J. Tigges, and J. G. Herndon. Ocular measurements throughout the adult life span of rhesus monkeys. *Investigative Ophthalmology & Visual Science*, 44(6):2373–2380, 2003.

[12] S. T. Fontana and R. F. Brubaker. Volume and depth of the anterior chamber in the normal aging human eye. *Archives of Ophthalmology*, 98(10):1803–1808, 1980.

[13] J. Herman and W. Usher. SALib: An open-source Python library for Sensitivity Analysis. *The Journal of Open Source Software*, 2(9), jan 2017.

[14] A. Hughes. A schematic eye for the rat. *Vision Research*, 19(5):569–588, 1979.

[15] L. Hutton-Smith. *Modelling the Pharmacokinetics and Pharmacodynamics of Macromolecules for the Treatment of Wet AMD.* PhD thesis, University of Oxford, 2018.

[16] L. A. Hutton-Smith, E. A. Gaffney, H. M. Byrne, P. K. Maini, D. Schwab, and N. A. Mazer. A mechanistic model of the intravitreal pharmacokinetics of large molecules and the pharmacodynamic suppression of ocular vascular endothelial growth factor levels by ranibizumab in patients with neovascular age-related macular degeneration. *Molecular Pharmaceutics*, 13(9):2941–2950, 2016.

[17] T. Iwanaga, W. Usher, and J. Herman. Toward SALib 2.0: Advancing the accessibility and interpretability of global sensitivity analyses. *Socio-Environmental Systems Modelling*, 4:18155, May 2022.

[18] H. Kaplan, C.-W. Chiang, J. Chen, and S.-K. Song. Vitreous volume of the mouse measured by quantitative high-resolution MRI. *Investigative Ophthalmology & Visual Science*, 51(13):4414–4414, 2010.

[19] E. A. Kearsley and J. T. Fong. Linearly independent sets of isotropic cartesian tensors of ranks up to eight. *Journal of Research of the National Bureau of Standards*, pages 49–58, 1975.

[20] J. F. Koretz, S. A. Strenk, L. M. Strenk, and J. L. Semmlow. Scheimpflug and high-resolution magnetic resonance imaging of the anterior segment: a comparative study. *J Opt Soc Am A Opt Image Sci Vis*, 21(3):346–354, 2004.

[21] J. Liu and H. Farid. Twenty-four-hour change in axial length in the rabbit eye. *Investigative Ophthalmology & Visual Science*, 39(13):2796–2799, 1998.

[22] D. C. Lozano and M. D. Twa. Development of a rat schematic eye from in vivo biometry and the correction of lateral magnification in SD-OCT imaging. *Investigative Ophthalmology & Visual Science*, 54(9):6446–6455, 2013.

[23] LUCENTIS. [package insert], South San Francisco, CA: Genentech, Inc, 2024.

[24] F. Manns, J.-M. Parel, D. Denham, C. Billotte, N. Ziebarth, D. Borja, V. Fernandez, M. Aly, E. Arrieta, A. Ho, et al. Optomechanical response of human and monkey lenses in a lens stretcher. *Investigative Ophthalmology & Visual Science*, 48(7):3260–3268, 2007.

[25] S. Marino, I. B. Hogue, C. J. Ray, and D. E. Kirschner. A methodology for performing global uncertainty and sensitivity analysis in systems biology. *Journal of Theoretical Biology*, 254(1):178–196, 2008.

[26] R. W. Massof and F. W. Chang. A revision of the rat schematic eye. *Vision Research*, 12(5):793–796, 1972.

[27] T. Mayhew and D. Astle. Photoreceptor number and outer segment disk membrane surface area in the retina of the rat: stereological data for whole organ and average photoreceptor cell. *Journal of Neurocytology*, 26(1):53–61, 1997.

[28] P. S. Muether, M. M. Hermann, K. Dröge, B. Kirchhof, and S. Fauser. Long-term stability of vascular endothelial growth factor suppression time under ranibizumab treatment in age-related macular degeneration. *American Journal of Ophthalmology*, 156(5):989–993, 2013.

[29] Y. Niwa, M. Kakinoki, T. Sawada, X. Wang, and M. Ohji. Ranibizumab and aflibercept: intraocular pharmacokinetics and their effects on aqueous VEGF level in vitrectomized and nonvitrectomized macaque eyes. *Investigative Ophthalmology & Visual Science*, 56(11):6501–6505, 2015.

[30] E. P. Osuobeni. Ocular components values and their intercorrelations in Saudi Arabians. *Ophthalmic and Physiological Optics*, 19(6):489–497, 1999.

[31] X. Pan, E. R. Muir, C. Sellitto, K. Wang, C. Cheng, B. Pierscionek, P. J. Donaldson, and T. W. White. Age-dependent changes in the water content and optical power of the in vivo mouse lens revealed by multi-parametric MRI and optical modeling. *Investigative Ophthalmology & Visual Science*, 64(4):24–24, 2023.

[32] J. Pe'er, M. Muckarem, and G. Zajicek. Epithelial cell migration in the normal rat lens. *Annals of Anatomy-Anatomischer Anzeiger*, 178(5):433–436, 1996.

[33] A. Reichenbach, J. Schnitzer, A. Friedrich, W. Ziegert, G. Brückner, and W. Schober. Development of the rabbit retina: I. size of eye and retina, and postnatal cell proliferation. *Anatomy and Embryology*, 183:287–297, 1991.

[34] A. M. Rosen, D. B. Denham, V. Fernandez, D. Borja, A. Ho, F. Manns, J.-M. Parel, and R. C. Augusteyn. In vitro dimensions and curvatures of human lenses. *Vision Research*, 46(6-7):1002–1009, 2006.

[35] A. Saltelli and R. Bolado. An alternative way to compute fourier amplitude sensitivity test (fast). *Computational Statistics & Data Analysis*, 26(4):445–460, 1998.

[36] A. Saltelli, S. Tarantola, and K.-S. Chan. A quantitative model-independent method for global sensitivity analysis of model output. *Technometrics*, 41(1):39–56, 1999.

[37] T. Sawada, J. Nakamura, Y. Nishida, K. Kani, S. Morikawa, and T. Inubushi. Magnetic resonance imaging studies of the volume of the rabbit eye with intravenous mannitol. *Current Eye Research*, 25(3):173–177, 2002.

[38] C. Schmucker and F. Schaeffel. In vivo biometry in the mouse eye with low coherence interferometry. *Vision Research*, 44(21):2445–2456, 2004.

[39] O. Sha and W. Kwong. Postnatal developmental changes of vitreous and lens volumes in sprague-dawley rats. *Neuroembryology and Aging*, 4(4):183–188, 2006.

[40] B. G. Short. Safety evaluation of ocular drug delivery formulations: techniques and practical considerations. *Toxicologic Pathology*, 36(1):49–62, 2008.

[41] T. V. Tkatchenko, Y. Shen, and A. V. Tkatchenko. Analysis of postnatal eye development in the mouse with high-resolution small animal magnetic resonance imaging. *Investigative Ophthalmology & Visual Science*, 51(1):21–27, 2010.

[42] I. Tsiapa, M. K. Tsilimbaris, E. Papadaki, P. Bouziotis, I. G. Pallikaris, A. H. Karantanas, and T. G. Maris. High resolution mr eye protocol optimization: Comparison between 3d-ciss, 3d-psif and 3d-vibe sequences. *Physica Medica*, 31(7):774–780, 2015.

[43] L. Werner, J. Chew, and N. Mamalis. Experimental evaluation of ophthalmic devices and solutions using rabbit models. *Veterinary Ophthalmology*, 9(5):281–291, 2006.

[44] K. C. Wikler, R. W. Williams, and P. Rakic. Photoreceptor mosaic: number and distribution of rods and cones in the rhesus monkey retina. *Journal of Comparative Neurology*, 297(4):499–508, 1990.

[45] J. Zhou, Y. Tu, Q. Chen, and W. Wei. Quantitative analysis with volume rendering of pathological myopic eyes by high-resolution three-dimensional magnetic resonance imaging. *Medicine*, 99(42), 2020.
